# Comparative Study of Bis-Schiff Case Containing Conjugated Oligomers Based on Phosphate and Silane Moieties: Investigation of Photophysical and Thermal Properties

**DOI:** 10.1021/acsomega.4c01403

**Published:** 2024-05-28

**Authors:** Feyza Kolcu, Süleyman Çulhaoğlu, İsmet Kaya

**Affiliations:** † Department of Chemistry, Polymer Synthesis and Analysis Lab., Çanakkale Onsekiz Mart University, Çanakkale 17020, Turkey; ‡ Lapseki Vocational School, Department of Chemistry and Chemical Processing Technologies, Çanakkale Onsekiz Mart University, Çanakkale 178, Turkey; § Barem Packaging Industry and Trade A.S., Tire 35910, İzmir, Turkey

## Abstract

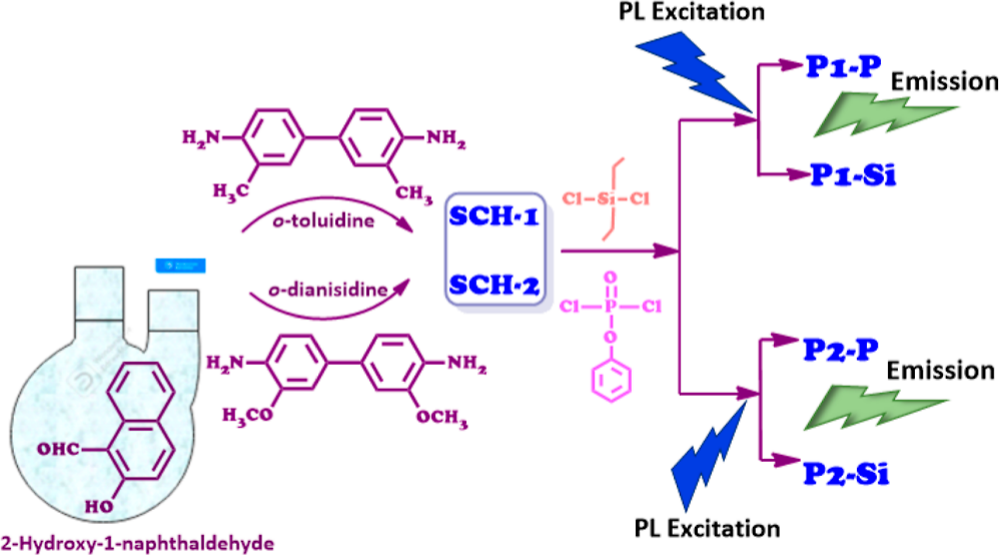

Oligo(azomethine)s bearing phosphate and silane moieties were the subject of an investigation within this study. The initial stage involved the synthesis of two Schiff base monomers, denoted as SCH-1 and SCH-2 (SCHs), each possessing a pair of hydroxyl functional groups. This was achieved through a loss of water between the aldehyde and diamine precursors. Subsequently, the Schiff base entities were subjected to oligomerization through HCl-mediated elimination due to the interaction between the hydroxyl groups of the Schiff bases and the chlorine moieties of dichlorodiethylsilane (Si) or phenyl dichlorophosphate (P). This procedure yielded distinct P-oligo(azomethine) (P1–P, P2–P) and Si-oligo(azomethine) (P1–Si and P2–Si) structures corresponding to each precursor. The molecular structures of the synthesized Schiff base monomers and oligo(azomethine)s were elucidated employing Fourier transform infrared, ^1^H NMR, and ^13^C NMR techniques. Thermal properties of the resulting products were assessed by utilizing thermogravimetric analysis (TG-DTG/DTA and DSC) techniques. Scanning electron microscopy (SEM) was employed to acquire high-resolution images and detailed surface information on the samples. Additionally, X-ray diffraction was employed to analyze the phase properties of the solid samples. Furthermore, the optical band gap (*E*_g_) values of the resulting P-oligo(azomethine)s and Si-oligo(azomethine)s were determined utilizing UV–vis spectrophotometer. The relatively low band gap values exhibited by the synthesized oligo(azomethine)s were indicative of their potential suitability as semiconductive materials in the realm of electronic and optoelectronic device fabrication. Photoluminescence (PL) measurements disclosed a green emission profile upon excitation by blue light. The oligo(azomethine)s incorporating methoxy groups demonstrated a red shift in comparison to their counterparts with methyl groups. Remarkably, no discernible fluctuations in fluorescence were observed over a 3600 s interval under consistent conditions. This observation underscored the inherent stability of the PL emission across the spectral range of exciting light. Thermal analyses unveiled high thermal stability of the synthesized oligo(azomethine)s, sustaining their structural integrity up to 220 °C. The char % of P-oligo(azomethine)s and Si-oligo(azomethine)s were observed to fall within the range of 29.45–55.47% at 1000 °C. SEM images revealed the absence of pores on the surface of P2–Si, which exhibited the highest limiting oxygen index and thermal heat release index (*T*_HRI_) values.

## 1 Introduction

The progress of novel polymeric materials exhibiting exceptional combined characteristics, necessary to fulfill escalating requisites while concurrently minimizing production expenditures, constitutes a pivotal subject within the domain of polymer science.^1^ The attention of numerous research groups has been captivated by π-conjugated polymers due to their potential across various domains, including industrial and technological applications.^2^ An additional significant category within the field of high-performance π-conjugated polymers encompasses poly(azomethine)s derived from Schiff bases.^2−4^

The pronounced allure surrounding these conjugated polymers stems from their commendable attributes encompassing thermal stability, mechanical robustness, optoelectronic qualities, photovoltaic potential, paramagnetism, electrical conductance, liquid crystalline behavior, therapeutic functionality, and their propensity to form metal complexes.^5−9^ These attributes render poly(azomethine)s suitable for sensor applications and light-emitting materials. Furthermore, diversely modified iterations of poly(azomethine)s, such as poly(azomethine-ether)s,^10,11^ poly(azomethine-urethane)s,^12^ poly(azomethine-sulfone)s,^13^ and poly(azomethine-carbonate)s,^14^ have been synthesized to enhance the solubility and processability of these distinctive polymer variants.

Poly(azomethine)s have emerged as prominent subjects of investigation owing to their intricate optical and electronic characteristics and have been extensively reviewed in the context of optoelectronic appliances with the inclusion of polymer light-emitting diodes and polymer solar cells.^15−17^ The isoelectronic character of the azomethine (imine, –HC=N) bonds present in oligo(azomethine)s or poly(azomethine)s has engendered similar optoelectronic performance attributes across their corresponding polymers,^18,19^ exemplified by poly(*p*-phenylenevinylene),^20^ a material amenable to fine-tuning its properties to manifest a notable fluorescence quantum yield upon doping, alongside robust chemical and electrochemical resistance.^21−25^ Poly(azomethine)s offer a host of advantages, as they can be synthesized via metal-free polycondensation with water as the sole byproduct.^26−29^

Despite their remarkable characteristics, aromatic poly(azomethine)s exhibit limited solubility in organic solvents and elevated melting temperatures, thereby constituting significant impediments for their viable industrial applications. Incorporating polar groups or flexible units like siloxane units into the conjugated aromatic azomethine frameworks presents an alternative way for enhancing their attributes.^8,29,30^ Ester or ether linkage-containing poly(siloxane-azomethine)s have been appeared in the literature.^31−34^ Furthermore, it is widely recognized that flame retardants incorporating silicon offer the distinct advantage of exceptional chemical stability and elevated resistance to high temperatures.^35,36^ In addition, silicon- and phosphorus-incorporating flame retardants are recognized as environmentally benign flame retardant agents, offering an alternative choice for effectively augmenting the thermal stability of materials.^37−40^ Due to the synergistic interaction between phosphorus and silicon elements, compounds incorporating phosphorus have been recognized as one of the potent flame retardants in many recent investigations.^41−43^ Besides, the inclusion of phosphorus within the polymer matrix has the potential to enhance solubility and thermal stability.^44−47^

Phenyl dichlorophosphate, which features a phosphorus atom and a benzene ring, exhibits elevated thermal stability and is capable of engaging in reactions with monomers containing hydroxyl functionalities,^48,49^ enabling the synthesis of phosphorus-containing flame-retardant monomers that confer exceptional flame-retardant characteristics upon polymers.^50−52^ During the thermal degradation process, silicon carbide functions to protect the polymer residue from further decomposition at elevated temperatures. This protection arises from the presence of reaction products of silicon that persist on the surface of the char, ultimately leading to the formation of a protective silica layer. In exposure to flames, the inclusion of phosphorus encourages the propensity for char formation, while silicon augments the char’s thermal stability.^53−55^

This study primarily aimed at the synthesis of two oligo(azomethine)s featuring phosphate and silane units incorporated within the oligo(azomethine) backbone. A diverse array of characterization techniques was employed to examine the influence of –HC=N- binding within the oligo(azomethine) structures on the inherent physical, optical, thermal, and morphological attributes of the resultant oligo(azomethine)s. It is hypothesized that the obtained oligo(azomethine)s, founded upon silane and phosphate constituents, are likely to exhibit not only commendable fluorescence properties but also heightened thermal stability.

## 2 Experimental Section

### 2.1 Materials

2-Hydroxy-1-naphthaldehyde, *o*-tolidine, *o*-dianisidine, *N*,*N*-dimethylformamide (DMF), dimethyl sulfoxide (DMSO), ethanol (EtOH), tetrahydrofuran (THF), methanol (CH_3_OH), *n*-hexane, acetonitrile (CH_3_CN), toluene, dichloromethane (CH_2_Cl_2_), *N*,*N*-dimethylacetamide (DMAc), chloroform, anhydrous sodium carbonate (Na_2_CO_3_) dichlorodiethylsilane, and phenyl dichlorophosphate were procured from Merck Chemical Co. (Germany). Tetra-*n*-butylammoniumhexafluorophosphate [(CH_3_CH_2_CH_2_CH_2_)_4_N(PF_6_)), 98%] was sourced from Aldrich Chemistry (Switzerland). All of the reagents were used without further purification.

### 2.2 Synthesis of Schiff Base Monomers (SCH-1 and SCH-2)

SCH-1,[1,1′-(((3,3′-dimethyl-[1,1′-biphenyl]-4,4′-diyl)bis(azanylylidene))bis (methanylylidene))bis(naphthalen-2-ol)] was synthesized using 2-hydroxy-1-naphthaldehyde (0.5165 g, 3.0 mmol) dissolved in 30 mL of ethanol, followed by the addition of *o*-tolidine (0.3184 g, 1.5 mmol) to this solution. The mixture was refluxed at 70 °C for 1 h. After 5 min, the product settled in the reaction medium.

**Scheme 1:**
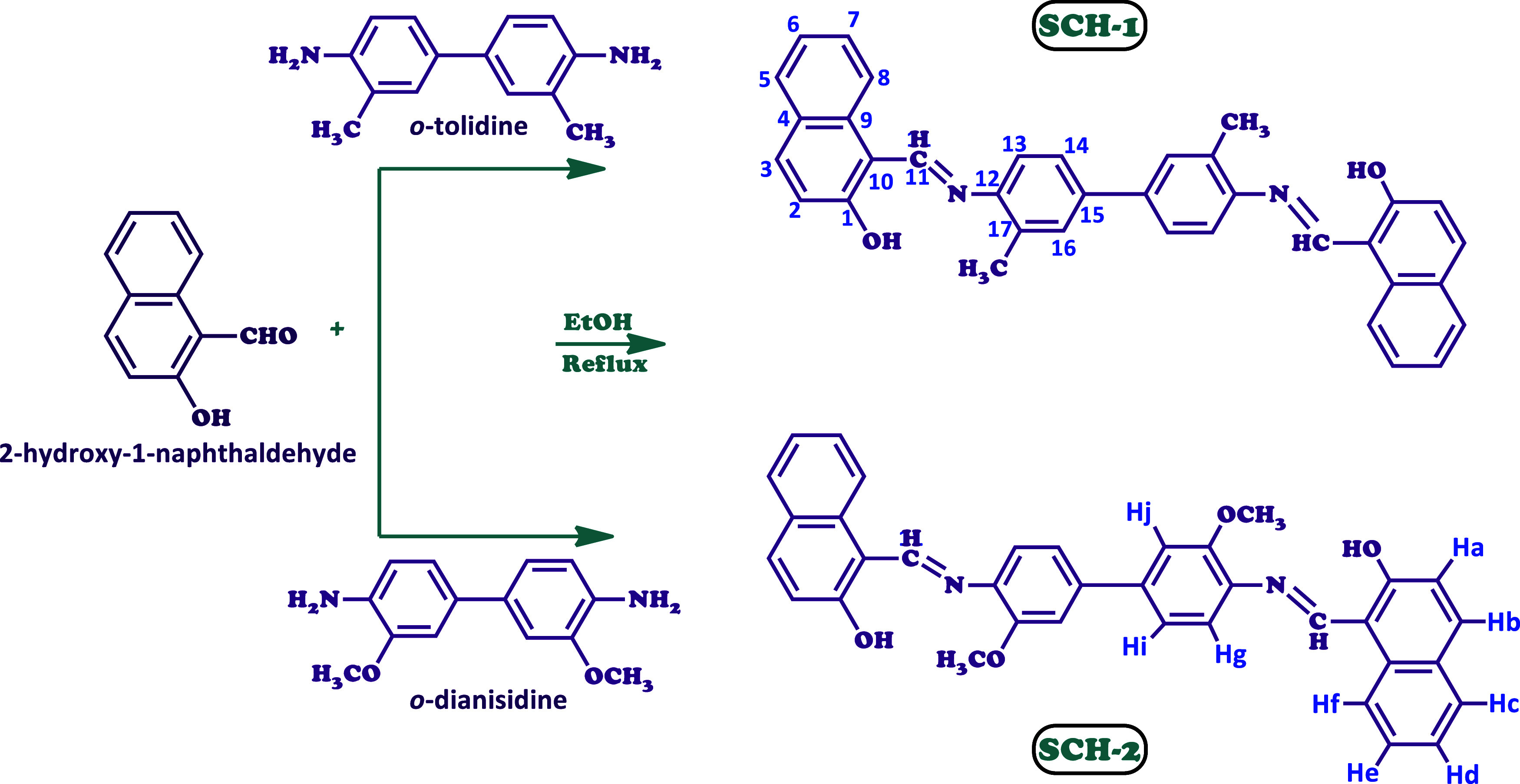
Synthesis of Schiff Bases 1 and 2 (SCH-1 and SCH-2)

SCH-2, [1,1′-(((3,3′-dimethoxy-[1,1′-biphenyl]-4,4′-diyl)bis(azanylylidene)) bis(methanylylidene))bis(naphthalen-2-ol)] was synthesized using 2-hydroxy-1-naphthaldehyde (0.5165 g, 3.0 mmol) dissolved in ethanol (30 mL), and then *o*-dianisidine (0.3664 g, 1.5 mmol) was added to this solution. The mixture was refluxed at 70 °C for 1 h. After 5 min, the product settled in the reaction medium. The products (SCH-1 and SCH-2) were purified via crystallization in ethanol. The yields of SCH-1 and SCH-2 were calculated as 80 and 86%, respectively. The synthesis pathways of Schiff bases 1 and 2 (SCHs) can be observed in Scheme 1.

#### 2.2.1 SCH-1: FT-IR (cm^–1^)

3358 (Ar–O–H), 3022 (C–H, aromatic), 1611 (HC=N, imine), 1540, 1481 (C=C, aromatic), 1291 (C–N), and 1205 (Ar–C–O). ^1^H NMR (DMSO-*d*_6_): δ ppm; 9.73 (s, 2Ar–OH), 8.54 (s, 2CH=N–), 7.99 (d, 2H, Hb), 7.94 (d, 2H, Hf), 7.77 (d, 2H, Hc), 7.55 (t, 2H, He), 7.34 (t, 2H, Hd), 7.28 (s, 2H, Hj), 7.21 (d, 2H, Hi), 7.19 (d, 2H, Hg), 7.11 (d, 2H, Ha), 2.17 (s, 6H, -CH_3_). ^13^C NMR (DMSO-*d*_6_): δ ppm, 162.33 (C11), 154.36 (C1), 137.45 (C15), 133.69 (C12), 129.18 (C3), 128.52 (C16), 128.28 (C17), 126.86 (C4), 125.65 (C5), 125.21 (C6), 124.49 (C7), 123.55 (C14), 120.78 (C2), 118.66 (C8), 109.55 (C10), 17.88 (-CH_3_).

#### 2.2.2 SCH-2: FT-IR (cm^–1^)

3358 (Ar–O–H), 3058 (C–H, aromatic), 1612 (HC=N, imine), 1540, 1481 (C=C, aromatic), 1294 (C–N), 1208 (Ar–C–O). ^1^H NMR (DMSO-*d*_6_): δ ppm; 9.55 (s, 2Ar–OH), 8.41 (s, 2CH=N-), 8.00 (2H, Hb), 7.82 (d, 2H, Hc), 7.69 (d, 2H, Hf), 7.50 (t, 2H, He), 7.31 (t, 2H, Hd), 7.15 (d, 2H, Hg), 6.95 (s, 2H, Hj), 6.81 (d, 2H, Hi), 6.62 (d, 2H, Ha), 3.81 (s, 6H, -OCH_3_). ^13^C NMR (DMSO-*d*_6_): δ ppm, 162.55 (C11), 150.83 (C1), 149.87 (C17), 147.08 (C15), 140.24 (C12), 137.91 (C9), 136.48 (C3), 134.14 (C16), 129.72 (C5), 128.52 (C4), 127.83 (C7), 120.34 (C2), 119.79 (C14), 118.15 (C6), 114.38 (C13), 109.26 (C8), 108.52 (C10), 55.69 (–OCH_3_).

### 2.3 Synthesis of P-Oligo(azomethine)s (P1–P and P2–P) and Si-Oligo(azomethine)s (P1–Si and P2–Si)

**Scheme 2:**
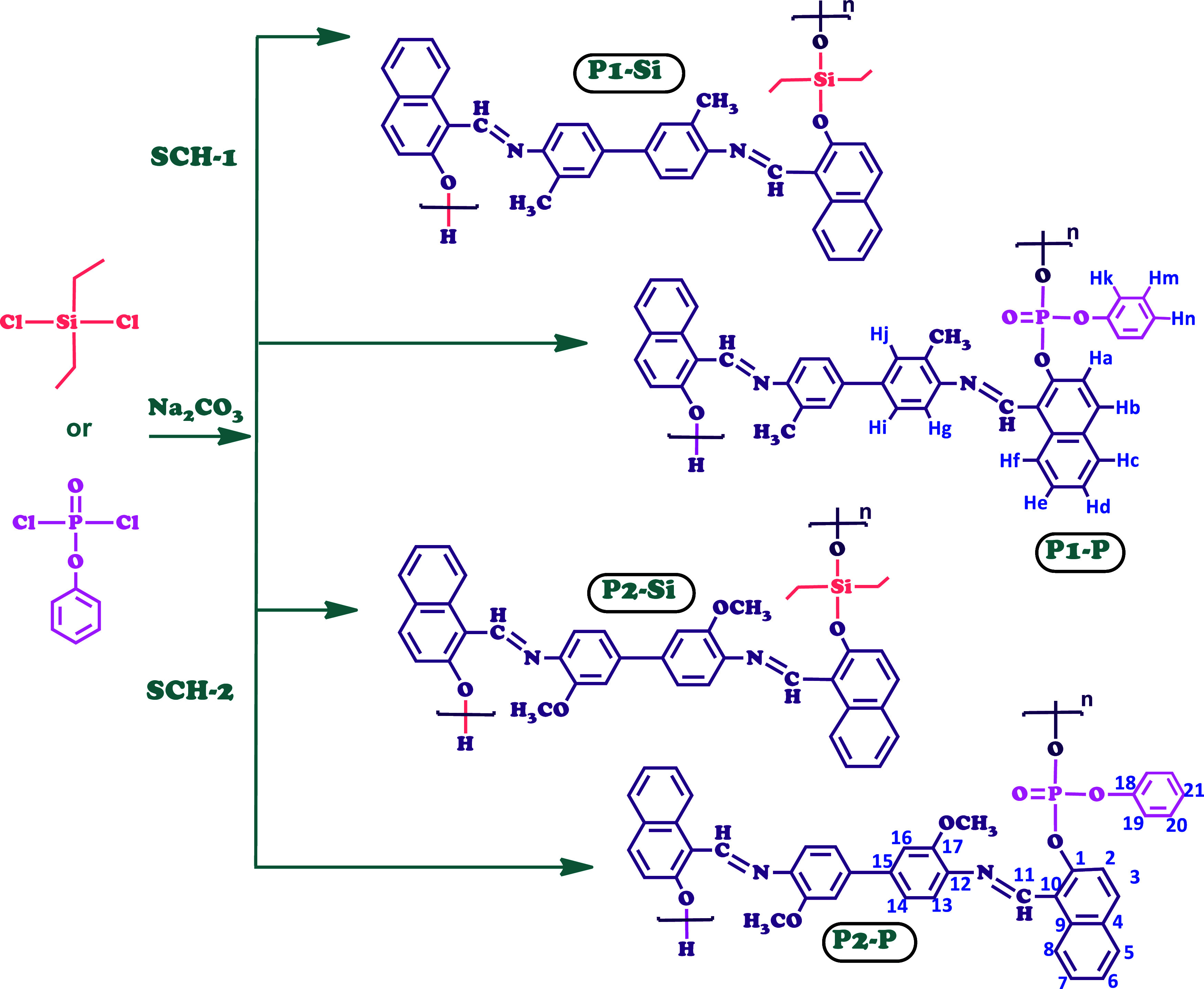
Synthesis of P-Oligo(azomethine)s (P1–P and P2–P) and Si-Oligo(azomethine)s (P1–Si and P2–Si)

The path for the synthesis of oligo(azomethine)s is given in Scheme 2. Initially SCH-1 (0.3484 g, 0.00067 mol) was dissolved in 25 mL of DMF in two separate 100 mL flasks. To each flask was added Na_2_CO_3_ (0.142 g, 0.00134 mol) in 5 mL of DMF. The reaction mixtures were then refluxed under argon gas at 160 °C for a duration of 1 h. Sequentially, phenyl dichlorophosphate (0.1 mL, 0.1412 g, 0.00067 mol) and dichlorodiethylsilane (0.1 mL, 0.1412 g, 0.00067 mol) were added to the marked mixtures, respectively. The oligomerization was allowed to proceed for an additional 16 h at 160 °C under argon gas. Subsequently, the reaction solutions were cooled to ambient temperature and transferred into 200 mL of ice-cold water. Following this process, the pure products were allowed to settle. The resulting P1–P and P2–P were washed with ethanol (2 × 25 mL) and dried in a vacuum oven at 60 °C for 24 h. The same procedure was applied to obtain P1–Si and P2–Si, commencing with SCH-2 (0.3698 g, 0.00067 mol). Ultimately, Si-oligo(azomethine)s exhibited a brown hue, whereas P-oligo(azomethine)s displayed shades of orange. P1–P, P1–Si, P2–P, and P2–Si were obtained with yields of 45, 30, 58, and 79%, respectively. The synthetic routes of the oligo(azomethine)s are depicted in Scheme 2.

#### 2.3.1 P1–P: FT-IR (cm^–1^)

3364 (Ar–O–H), 3058 (C–H, aromatic), 2924 (C–H, aliphatic), 1620 (HC=N imine), 1543, 1487 (C=C, aromatic), 1314 (P=O), 1291 (C–N), and 1210 (Ar–C–O). ^1^H NMR (DMSO-*d*_6_): δ ppm, 9.71, 9.68 (s, terminal Ar–OH), 8.52 (s, –CH=N-), 7.98 (d, Hk), 7.91 (t, He), 7.89 (d, Hb), 7.76 (d, Hf), 7.74 (d, Hc), 7.66 (t, Hm), 7.62 (d, Hi), 7.54 (s, Hj), 7.33 (t, Hd), 7.29 (d, Hg), 6.99 (t, Hn), 6.68 (d, Ha), 2.12 (s, –CH_3_). ^13^C NMR (DMSO-*d*_6_): δ ppm, 172.52 (C11), 154.37 (C1), 153.66 (C18), 146.99 (C15), 141.58 (C12), 139.85 (C9), 137.63 (C3), 133.59 (C19), 130.79 (C17), 129.22 (C16), 128.50 (C4), 127.97 (C5), 126.93 (C6), 125.71 (C7), 123.64 (C14), 123.09 (C13), 121.69 (C2), 120.68 (C20), 118.39 (C8), 109.14 (C10), 18.40 (–CH_3_).

#### 2.3.2 P1–Si: FT-IR (cm^–1^)

3372 (Ar–O–H), 3064 (C–H, aromatic), 2960–2880 (−C–H, aliphatic), 1620 (HC=N, imine), 1548, 1487 (C=C aromatic), 1291 (C–N), 1243 (Si–C), 1207 (Ar–C–O), 1077, 1006 (Si–O). ^1^H NMR (DMSO-*d*_6_): δ ppm; 9.71, 9.68 (s, terminal Ar–OH), 8.51 (s, –CH=N–), 7.93 (t, He), 7.89 (d, Hb), 7.75 (d, Hf), 7.54 (d, Hc), 7.33 (s, Hj), 7.28 (d, Hi), 7.09 (d, Hg), 6.99 (t, Hd), 6.68 (d, Ha), 2.12 (s, -CH_3_), 0.91 (t, -Si-CH_2_CH_3_), 0.54 (m, −Si–CH_2_CH_3_). ^13^C NMR (DMSO-*d*_6_): δ ppm, 172.24 (C11), 153.56 (C1), 146.83 (C15), 139.76 (C12), 137.16 (C9), 133.57 (C3), 130.64 (C16), 129.44 (C17), 128.38 (C4), 127.87 (C5), 127.00 (C6), 124.96 (C7), 124.39 (C14), 123.73 (C13), 121.64 (C2), 118.21 (C8), 25.55 (–Si–CH_2_CH_3_), 18.45 (–CH_3_), 7.32 (–Si–CH_2_CH_3_).

#### 2.3.3 P2–P: FT-IR (cm^–1^)

3369 (Ar–O–H), 3062 (C–H, aromatic), 2963–2924 (C–H, aliphatic), 1609 (-HC=N– imine), 1537, 1587 (C=C, aromatic), 1323 (P=O), 1294 (C–N), and 1205 (Ar–C–O). ^1^H NMR (DMSO-*d*_6_): δ ppm, 9.77 (s, terminal Ar–OH), 8.43 (s, -CH=N-), 8.27 (d, Hk), 8.08 (d, Hb), 8.0 (d, Hf), 7.81 (t, He), 7.69 (d, Hc), 7.50 (s, Hj), 7.42 (d, Hi), 7.31 (t, Hd), 7.27 (t, Hm), 7.15 (t, Hn), 6.81 (d, Hg), 6.71 (d, Ha), 3.87 (s, –OCH_3_). ^13^C NMR (DMSO-*d*_6_): δ ppm, 177.65 (C11), 160.46 (C1), 150.83 (C18), 149.86 (C17), 146.85 (C15), 140.24 (C12), 138.13 (C9), 135.80 (C3), 134.14 (C19), 129.94 (C16), 129.41 (C4), 128.52 (C5), 126.41 (C6), 125.21 (C7), 123.55 (C21), 120.78 (C14), 119.80 (C20), 118.14 (C13), 115.59 (C2), 114.16 (C8), 109.27 (C10), 56.72 (–OCH_3_).

#### 2.3.4 P2–Si: FT-IR (cm^–1^)

3370 (Ar–O–H), 3062 (C–H, aromatic), 2963–2877 (C–H, aliphatic), 1614 (HC=N, imine), 1505, 1460 (C=C, aromatic), 1294 (C–N), 1240 (Si–C), 1210 (Ar–C–O), 1080, and 1006 (Si–O). ^1^H NMR (DMSO-*d*_6_): δ ppm; 9.54 (s, terminal Ar–OH), 8.41 (s, CH=N–), 7.91 (t, He), 7.81 (d, Hb), 7.68 (d, Hf), 7.43 (s, Hj), 7.31 (d, Hc), 7.15 (d, Hi), 6.95 (d, Hg), 6.71 (t, Hd), 6.63 (d, Ha), 3.81 (s, -OCH_3_), 0.90 (t, -Si-CH_2_CH_3_), 0.48 (m, −Si–CH_2_CH_3_). ^13^C NMR (DMSO-*d*_6_): δ ppm, 177.13 (C11), 157.59 (C1), 149.85 (C17), 146.86 (C15), 146.31 (C12), 143.09 (C9), 138.13 (C16), 136.25 (C3), 131.30 (C4), 130.16 (C5), 129.94 (C2), 128.96 (C7), 127.30 (C2), 123.77 (C6), 118.68 (C14), 114.38 (C13), 112.05 (C8), 108.75 (C10), 55.67 (-OCH_3_), 29.15 (–Si–CH_2_CH_3_), 8.83 (–Si–CH_2_CH_3_).

### 2.4 Instrumentation

The infrared spectra of the synthesized SCHs (SCH-1 and SCH-2), as well as their P-oligo(azomethine)s and Si-oligo(azomethine)s, were acquired using a Spectrum One FT-IR system (PerkinElmer, USA) equipped with an ATR attachment sampling unit in the frequency range from 4000 to 400 cm^–1^. To elucidate the molecular structure of the synthesized compounds, ^1^H NMR and ^13^C NMR spectra were recorded using an Agilent 600 and 150 MHz Premium Compact NMR magnet, respectively. These measurements were performed in DMSO-*d*_6_ at 25 °C, with TMS provided as the internal standard.

UV–vis absorption spectra of the synthesized SCHs (SCH-1 and SCH-2) and their oligo(azomethine)s in DMF, spanning the wavelength between 240 and 600 nm, were recorded on Analytikjena Specord 210 Plus spectrophotometer (UK) at 25 °C. The measurements were conducted by using a quartz spectrophotometer cell with a path length of 1.0 cm. Additionally, UV–vis absorption spectra of the synthesized oligo(azomethine)s were collected in DMSO. The energy difference between the ground state and the excited state can be measured by determining the onset of absorption (λ_onset_) from the low energy side of UV–vis spectra. Optical band gap values (*E*_g_) were determined using *E*_g_ = h.c/λ_onset_ and can be turned into 1242/λ_onset_ in electron volts.^56^ The photoluminescence (PL) properties of the synthesized SCHs and their corresponding oligo(azomethine)s were investigated in DMF using a Shimadzu RF-5301PC spectrofluorophotometer (Japan). The photoluminescence (PL) quantum yield, denoted as QY, represents photoluminescence features of the SCHs and their oligo(azomethine)s, using a standard solution of fluorescein in 0.1 M aqueous NaOH serving as the reference. Equation 1 assists in the calculation of QY values as follows^57,58^
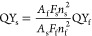
1where *A* represents the absorption at the maximum excitation wavelength, *F* signifies the integrated area beneath the fluorescence emission spectrum, and n denotes the refractive index of the solvent (DMF). The slit width values for the excitation and emission processes were maintained at 5 nm for both the sample and the standard.

The number-average molecular weight (Mn), weight-average molecular weight (Mw), and polydispersity index (PDI) values of the oligo(azomethine) samples (approximately 10 mg) were determined by employing a Gel Permeation Chromatography-Light Scattering (GPC-LS) instrument, specifically, the Malvern Viscotek GPC Dual 270 max (UK). The instrument was equipped with a Light Scattering (LS) and Refractive Index (RID) detector and a column dimension of 0.8 cm × 30 cm. An eluent of DMF containing 40 mM lithium bromide was employed, with a flow rate of 0.4 mL per minute. The calibration of the instrument was achieved utilizing a set of polystyrene standards with peak molecular weights ranging from 162 to 60,000 dalton (Da).

Thermal [TG (thermal gravimetric) and DTG (differential thermal gravimetric)] analyses for both SCHs and their respective oligo(azomethine)s were carried out utilizing a PerkinElmer Diamond Thermal Analyzer (USA) within a temperature range spanning from 25 °C to 1000 °C. A heating rate of 10 °C per minute was applied within a nitrogen gas environment maintaining a flow rate of 200 mL per minute. In order to determine the glass transition temperatures (*T*_g_) of the synthesized oligo(azomethine)s, differential scanning calorimetry (DSC) analysis was conducted employing a PerkinElmer Sapphire instrument (USA) within the temperature range between 25 and 420 °C, under a N_2_ atmosphere (100 mL min^–1^), with a heating rate of 10 °C per minute. TG and DSC measurements were carried out utilizing sealed aluminum pans, each containing approximately 5 mg of the sample.

The JSM-7100F Field Emission SEM (JEOL, Japan) provided detailed images of the surface of the synthesized oligo(azomethine)s in the study. Sputter coating process was used to make a thin gold/palladium film onto powder samples. X-ray diffraction (XRD) measurements were recorded by a PANalytical empyrean model X-ray diffractometer instrument (The Netherlands) with CuK_α_ radiation at a wavelength of 1.54 Å over a range of 2θ from 5 to 90° with a scanning rate of 4° min^–1^.

## 3 Results and Discussion

### 3.1 Solubility Testing of the Synthesized Compounds

Schiff bases 1 and 2 (SCHs) along with their corresponding P-oligo(azomethine)s and Si-oligo(azomethine)s were synthesized by following the procedures outlined in Schemes 1 and 2. The SCHs were obtained as the sole product with a notably high yield. SCH-1 exhibited a red hue, while SCH-2 exhibited orange color. Qualitative solubility assessments of the synthesized compounds were conducted by taking 1 mg of each sample per 1 mL of solvent at room temperature. The synthesized SCHs, P-oligo(azomethine)s (P1–P, P2–P), and Si-oligo(azomethine)s (P1–Si, P2–Si) exhibited solubility in CH_3_OH, CH_3_CN, DMF, DMSO, THF, DMAc, and chloroform. However, it is worth noting that their solubility in ethanol was partial. In contrast, all of the synthesized compounds demonstrated insolubility in hexane and toluene. The observed enhancement in solubility can be ascribed to the incorporation of phenyl dichlorophosphate and dichlorodiethylsilane moieties into the oligomer backbone. This modification increased the flexibility and polarity of the oligomers, mitigating the constraints imposed by the rigid planar π-conjugation exhibited by the repeating units containing naphthyl and phenyl groups in the oligo(azomethine)s.^59−61^

### 3.2 Assessment of FT-IR and NMR Measurements

**Figure 1:**
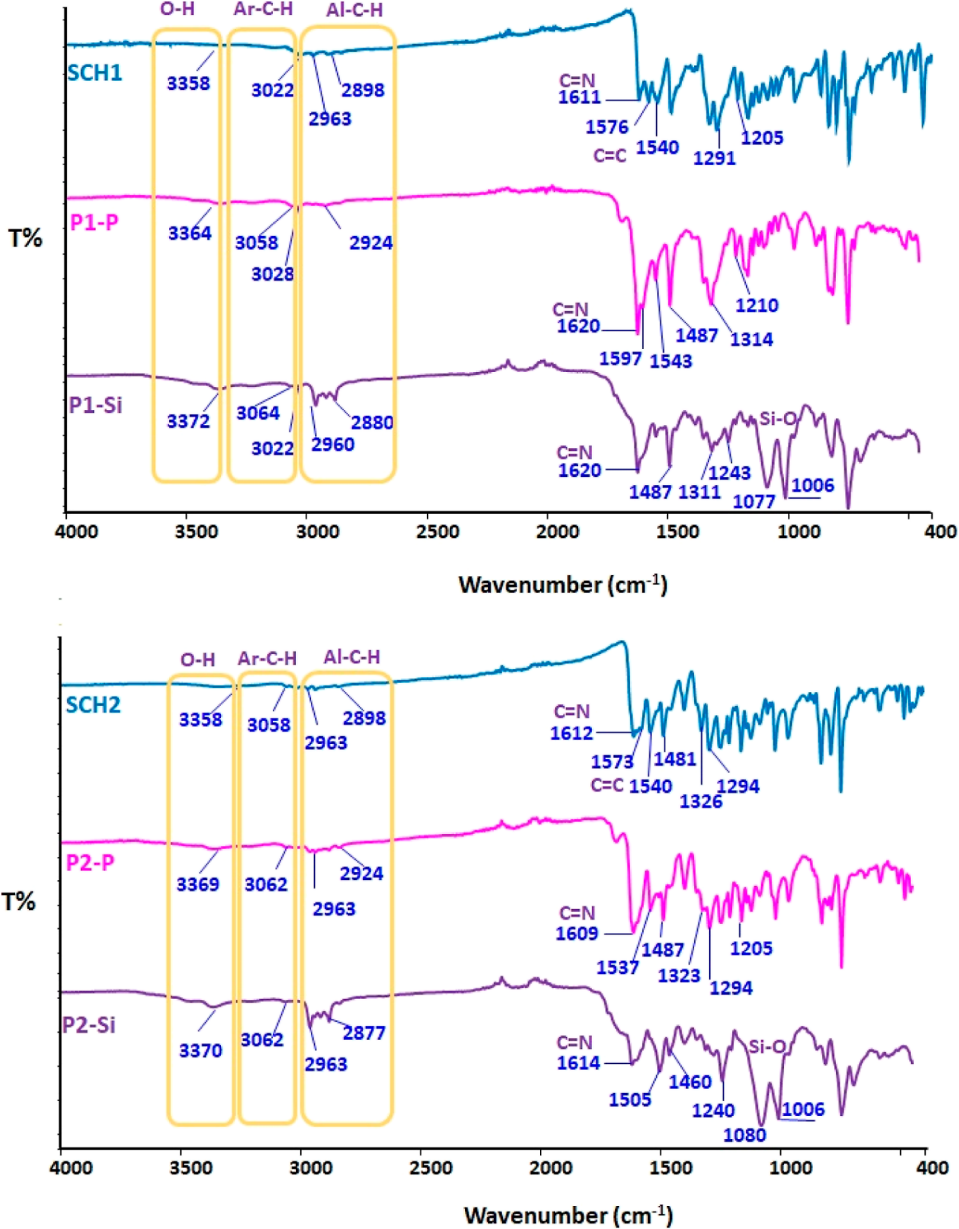
FT-IR spectra of SCH-1, SCH-2, P-oligo(azomethine)s (P1–P, P2–P), and Si-oligo(azomethine)s (P1–Si and P2–Si).

FT-IR spectra for both SCHs and their oligo(azomethine)s are depicted in Figure 1. In the FT-IR spectrum of the synthesized SCHs, the broad peaks seen in the proximity of 3358 cm^–1^ are attributed to the stretching vibrations of the phenolic OH groups. These vibrations underwent a frequency shift toward higher values by about 6 and 14 cm^–1^ due to the linkages of dichlorodiethylsilane and dichlorophosphate during the oligomerization. The vibrations associated with aromatic C–H stretching were discerned at 3058 and 3022 cm^–1^ for the SCHs and oligo(azomethine)s with values of 3064 and 3058 cm^–1^.

As shown in Figure 1, the vibrational band values for the imine (HC=N) bond of compounds SCH-1, P1–P, P1–Si, SCH-2, P2–P, and P2–Si were observed at 1611, 1620, 1620, 1612, 1609, and 1614 cm^–1^, respectively. This observation provided compelling evidence of imine (HC=N) bond formation in the synthesized compounds, indicating that the azomethine groups remained intact during oligomerization.^62^ The upshift observed in the C=N vibration frequencies within the oligomers may be attributed to the integration of dichlorodiethylsilane and dichlorophosphate groups, resulting in the formation of polar Si–O and P–O bonds. The confirmation of phenyl dichlorophosphate attachment to SCH-1 and SCH-2 was subsequently ascertained by the observation of P=O vibrational bands, which exhibited overlap with the Schiff base structural moieties. These vibrational bands manifested at wavenumbers of 1314 and 1323 cm^–1^ in the spectra of P1–P and P2–P, respectively.^63,64^ The signals at wavenumbers of 1291 and 1294 cm^–1^ for SCHs represented C–N stretching vibrations, while the peaks at frequencies of 1205 and 1208 cm^–1^, respectively, are attributed to Ar–C–O stretching vibrations. The confirmation of the attachment of phenyl dichlorophosphate to SB-1 and SB-2 was substantiated by the presence of two signals associated with P=O and P–O–C vibrations, manifesting at 1100 and 976 cm^–1^, respectively, in the P-oligo(azomethine)s.^63^ In the context of Si–O stretching vibrations with regard to P1–Si and P2–Si, the peaks observed at 1077 and 1006 cm^–1^ are ascribed to the attachment of dichlorodiethylsilane to SCH-1. Similarly, the peaks at 1080 and 1006 cm^–1^ are indicative of the attachment of dichlorodiethylsilane to SCH-2.^55^ Additionally, a newly emerged band at 1243 and 1240 cm^–1^, respectively, can also be ascribed to the stretching vibrations of Si–C bonds in P1–Si and P2–Si.^65^ As depicted in Figure 1, bands associated with aliphatic symmetric and asymmetric C–H stretching vibrations, resulting from the formation of bonds between SCHs and dichlorodiethylsilane, were seen in the wavenumber range from 2963 to 2880 cm^–1^. Furthermore, the existence of the strong phenolic C–O band at 1237 cm^–1^ and nonexistence of the NH band are other evidence for the phenol-imine form of the diimine in the solid state.

**Figure 2:**
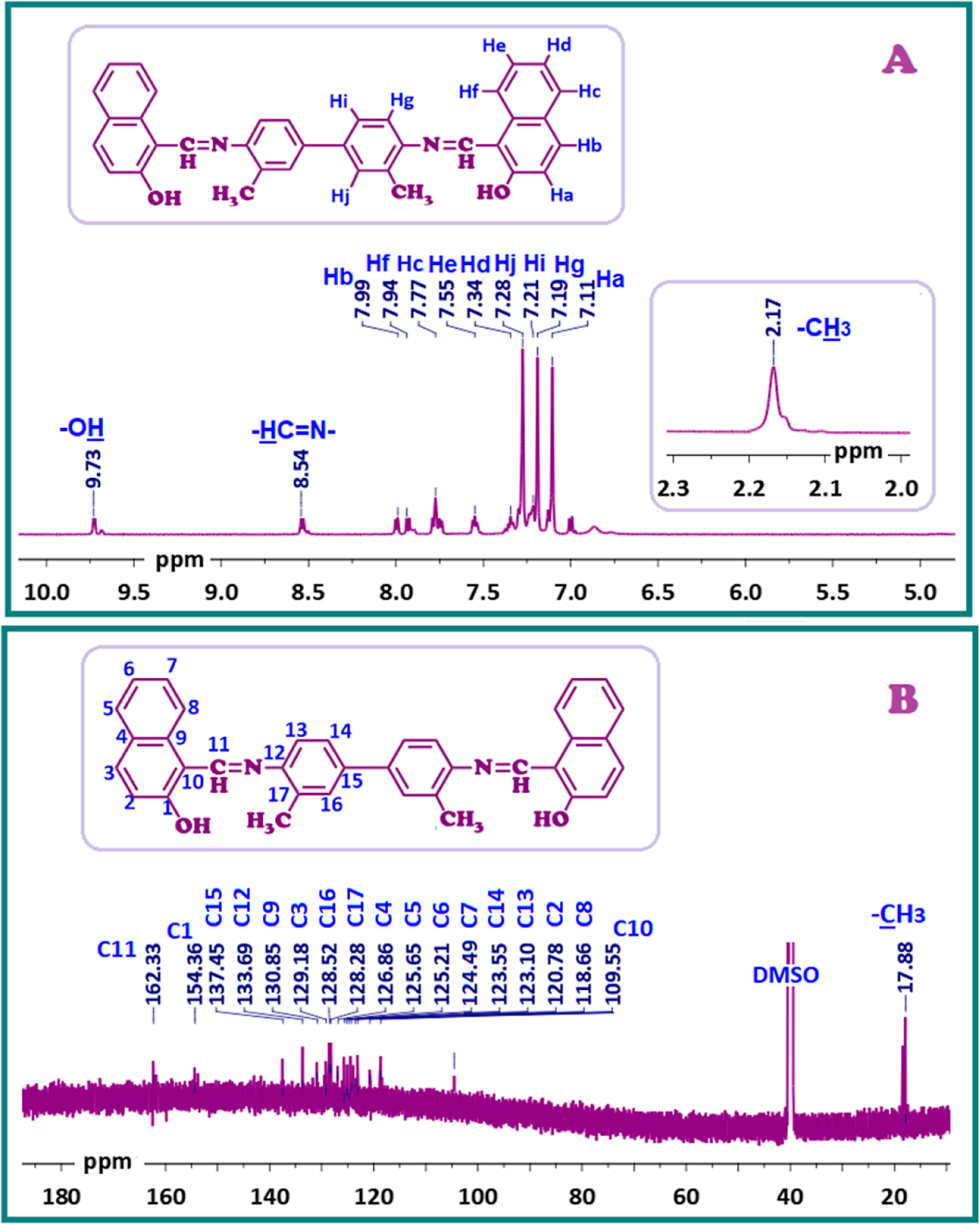
(A) ^1^H NMR and (B) ^13^C NMR spectra of SCH-1.

Structural characterization involved the verification of ^1^H- and ^13^C NMR spectra for the Schiff bases (SCH-1 and SCH-2) and the synthesized oligo(azomethine)s (P1–P, P1–Si, P2–P, and P2–Si). The NMR spectral data for the compounds were recorded in DMSO-*d*_6_ and the chemical shift values are presented in the experimental section. The ^1^H NMR and ^13^C NMR spectra of SCH-1 are depicted in Figure 2 as a model of a bis-azomethine. As shown in Figure 2A, the proton signals observed at 9.73 and 8.54 ppm were attributed to the -OH and -HC=N- functional groups of SCH-1, respectively. Similarly, for SCH-2, these proton signals were detected at 9.55 ppm and 8.41 ppm (see Figure S1A), in accordance with values reported in the literature.^62^ The presence of a peak assigned to the HC=N group in the spectrum confirmed the formation of the SCHs. The ratio of two protons of the -HC=N– group to two aromatic –OH protons being 1 provided evidence of symmetry in the structure of SCH-1.

In Figure 2B, 18 carbon signals corresponding to different chemical environments within SCH-1’s structure were observed. The signal assigned to the azomethine (–CH=N–) group at C11 and the signal related to the –OH group at C1 were seen at 162.33 and 154.36 ppm, respectively. The signals observed between 137.45 ppm and 109.55 ppm was related to the aromatic carbons between C1 and C17. The positive mesomeric (+M) effect of the –OH group, due to delocalization of the lone pair on the benzene ring, notably increased the electron density, particularly in the ortho and para positions. Consequently, the C3 signal, located at the meta position to the C1 carrying the –OH group, was observed at 129.18 ppm.

No observation was present for the hydroxyl proton far downfield at δ = 13.97–12.54 ppm due to extensive hydrogen bonding between the imine nitrogen (HC=N) and the OH group.^66^ Consequently, the Schiff base monomers depicted in Figures 2A and S1A exhibited the presence of free hydroxyl protons (OH) and azomethine protons (HC=N), indicative of their exclusive existence in the phenol-form, as elucidated.^67^ This deduction was further substantiated by the nonappearance of the quinoidal C=O signal (δ = 190–220 ppm)^68^ in the ^13^C NMR spectra of SCH-1 and SCH-2, as seen in Figure 2B and Figure S1B.

**Figure 3:**
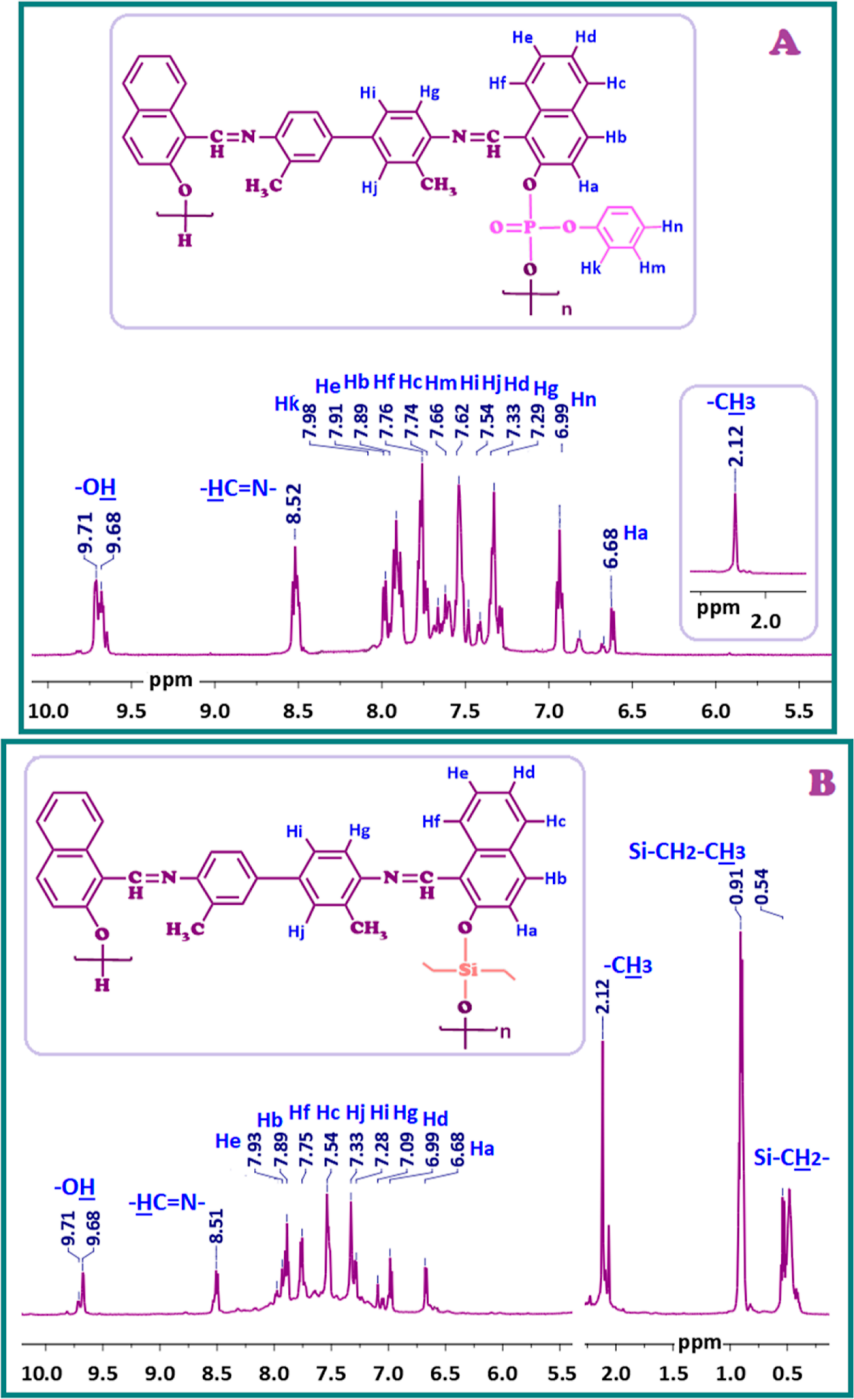
^1^H NMR spectra of (A) P1–P and (B) P1–Si.

**Figure 4:**
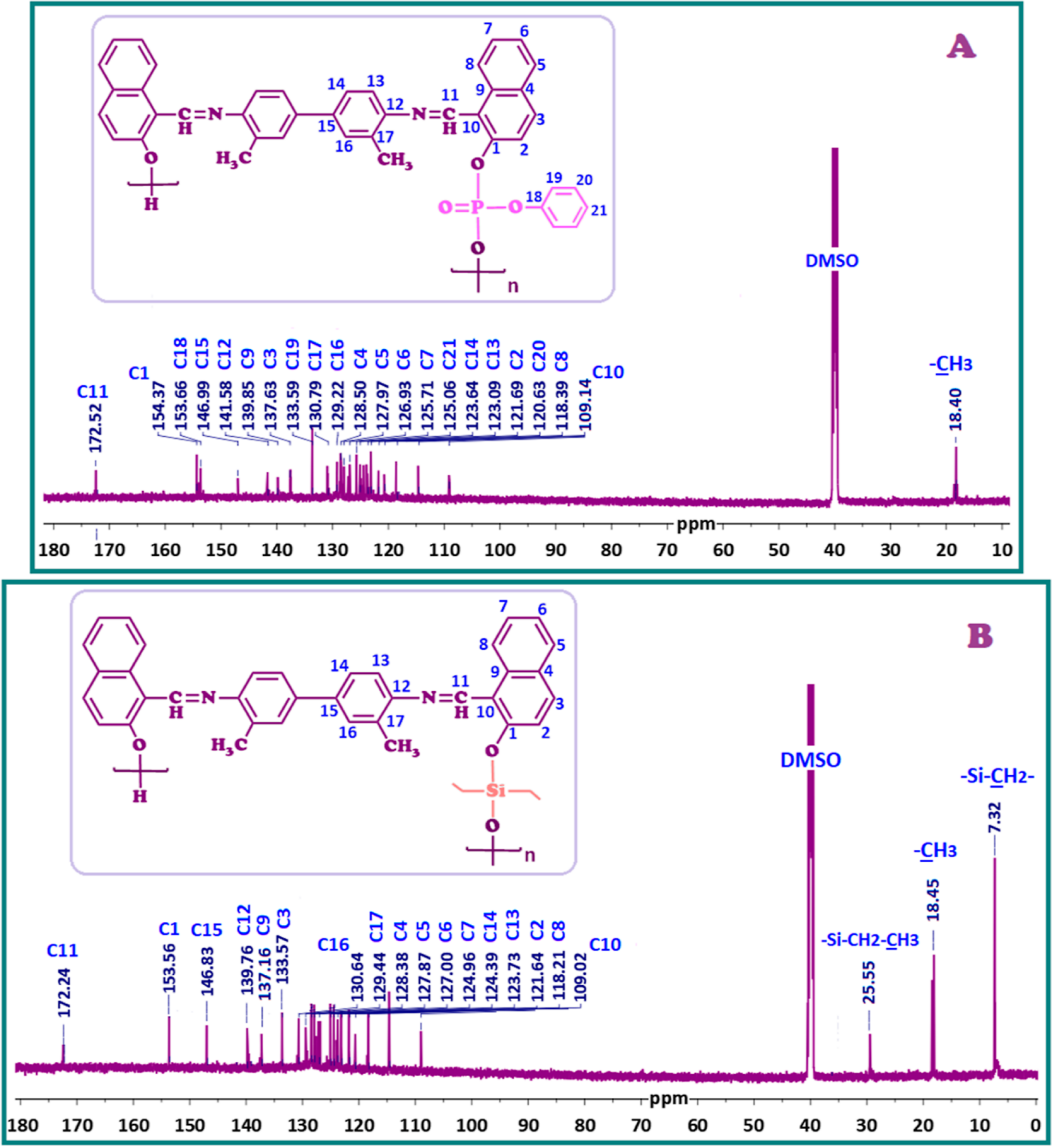
^13^C NMR spectra of (A) P1–P and (B) P1–Si.

Figures 3 and 4 display the ^1^H NMR and ^13^C NMR spectra of P1–P and P1–Si, respectively. The broad proton signals in the ^1^H NMR spectrum of the oligomer synthesis proved the presence of repeating units with distinct chemical environments in both the monomer and the two oligomers. The ^1^H NMR spectra of P1–P and P1–Si confirmed the presence of terminal -OH groups at 9.71 ppm, and –HC=N– protons at 8.52 and 8.51 ppm, respectively (Figure 3).

The equally large coupling constants between hydrogens separated by five bonds (*J*_1,5_) can be observed.^69,70^ Evidently, the phenomenon of orbital overlap between the π orbital associated with the double bond and the hybridized N=C–H orbital facilitates the nuclear interactions responsible for coupling with the proton of the OH group in SCH-1. This interaction consequently leads to the observed weak splitting of the protons associated with the OH and HC=N groups, as depicted in Figure 2A. Due to the oligomerization process, the molecular symmetry of SCH-1 and SCH-2 was disrupted, leading to the appearance of a double-peak for the terminal OH protons and undergoing splitting with a coupling constant of *J* = 0.03 Hz, for P1–P and P1–Si (Figure 3). Particularly, the imine proton signal in P1–Si experienced an upfield shift from 8.54 ppm in the monomer to 8.30 ppm, attributed to the influence of the more electropositive Si atom in the oligomer chain. The electron pair on the oxygen atom induced upfield shifts for Ha in P1–P and P1–Si at 6.68 ppm, as seen in Figure 3A,B.

The Hk, Hm, and Hn protons in P1–P, together with the other protons of SCH-1, were associated with phenyl dichlorophosphate binding. The Hk proton, located at the ortho position to the phosphate group, exhibited a signal at 7.98 ppm, lower in chemical shift compared to Hm at 7.66 ppm and Hn at 6.99 ppm (Figure 3A). The multiple peaks at 0.54 ppm and a triplet at 0.91 ppm pertained to Si–CH_2_– and Si–CH_2_–CH_3_, respectively. Aromatic proton signals for the oligo(azomethine)s were displayed between 7.98 ppm and 6.69 ppm, and between 7.93 ppm and 6.68 ppm (Figure 3B). Upon comparison of the spectra of the synthesized oligo(azomethine)s with the ^1^H NMR spectra of SCH-1, it was observed that the peaks corresponding to the oligomeric structure were broadened, indicating the existence of recurring phenyl units within the synthesized oligo(azomethine)s, each characterized by distinct chemical environments.

Based on the above interpretations, the proton signals seemed to be identical in the ^1^H NMR spectra of P2–P and P2–Si, as seen in Figures S2A and S3A, respectively. The proton signals of the -HC=N- functional group were detected at 8.42 and 8.59 ppm, respectively. The Hk, Hm, and Hn protons in P2–P, along with other protons of SCH-2, were associated with phenyl dichlorophosphate binding. The Hk proton, located at the ortho position to the phosphate group, exhibited a signal at 8.26 ppm, downfield in chemical shift compared to that of Hm at 7.27 ppm and Hn at 7.15 ppm. Consistent with the electronegativity difference between the C and Si atoms, multiple peaks at 0.48 ppm were observed for Si–CH_2_- and a triplet at 0.91 ppm was observed for Si–CH_2_–CH_3_.

The ^13^C NMR spectra of P1–P and P1–Si are displayed in Figure 4A,B, respectively. The carbon signals for the –HC=N- group in P1–P and P1–Si were determined based on the peaks seen at 170.52 ppm and 172.24 ppm, respectively. The C10 carbons in P1–P and P1–Si, located at the ortho position to C1, exhibited higher electron density due to electron release from the oxygen atom, resulting in carbon signals at 109.14 ppm and 109.02 ppm, respectively (Figure 4A). Additionally, the signals for the aromatic carbon atoms numbered as C1 and C21 in P1–P were observed between 154.37 and 118.39 ppm. The carbon signal attributed to –CH_3_ was observed at 18.40 ppm. In the case of P1–Si, the aromatic carbon signals were observed between 153.56 and 118.21 ppm (Figure 4B). Due to silicon’s lower electronegativity compared to carbon, the methyl-bound silicon groups in P1–Si, Si–CH_2_–, and Si–CH_2_–CH_3_, shifted further upfield, appearing at 7.32 ppm and 25.55 ppm, respectively. The chemical shift of the carbon atom signal related to the –CH_3_ group in P1–Si was ascertained to be 18.45 ppm (Figure 4B).

As seen in Figures S2B and S3B, respectively, the carbon signals for P2–P and P2–Si exhibited similarities to those observed in P1–P and P1–Si. The carbon signals for the imine (–CH=N–) carbons in P2–P and P2–Si, designated as C11, were observed at 177.65 and 177.24 ppm, respectively. The carbon atoms C18, C19, C20, and C21 in P2–P were associated with the binding of phenyl dichlorophosphate, along with other SCH-2 carbons. Additionally, the signals for the aromatic carbon atoms numbered as C1 and C21 in P1–P were observed between 160.56 and 109.89 ppm. The carbon signal attributed to -OCH_3_ as C17 appeared at 56.72 ppm. In P2–Si, the carbon atoms associated with –CH_2_–CH_3_ groups bound to silicon were observed at 7.48 and 29.30 ppm, respectively. The other aromatic carbons of P2–Si, numbered as C1 and C17, exhibited signals between 157.65 and 108.76 ppm.

### 3.3 GPC Results of P-Oligo(azomethine)s and Si-Oligo(azomethine)s

The GPC analysis of the oligomers assisted in determining the molecular weights of P- oligo(azomethine)s and Si-oligo(azomethine)s. Table 1 presents a synopsis of the gel permeation chromatography (GPC) chromatogram analysis results for the synthesized oligomers. The Mn and Mw values were calculated as follows: 4850 and 5400 Da; 4650 and 5100 Da; 5500 and 6300 Da; and 5000 and 5600 Da were detected for P1–P, P2–P, P1–Si, and P2–Si, respectively, exhibiting oligomeric structure. The PDI values were calculated by taking the ratio of Mw to Mn, resulting in values of 1.11, 1.10, 1.11, and 1.11 for P1–P, P2–P, P1–Si, and P–Si, respectively. These findings indicated a high level of homogeneity in the samples with particles demonstrating a notably consistent size distribution. Consequently, considering the relationship between Mn and Mw, the estimated average number of repeating units for the synthesized oligomers falls within the range of approximately 4–5.

**Table 1:** GPC Analysis Results for P-Oligo(azomethine)s and Si-Oligo(azomethine)s

	Mn (Da)	Mw (Da)	PDI = *M*_w_/*M*_n_	Mz (Da)	Mp (Da)
P1–Si	5100	5650	1.108	6300	3400
P1–P	4850	5400	1.113	5550	2800
P2–Si	5200	5800	1.115	6900	3100
P2–P	5400	5950	1.102	6700	3650

### 3.4 Optical Properties

#### 3.4.1 UV–vis Absorption Spectra

**Figure 5:**
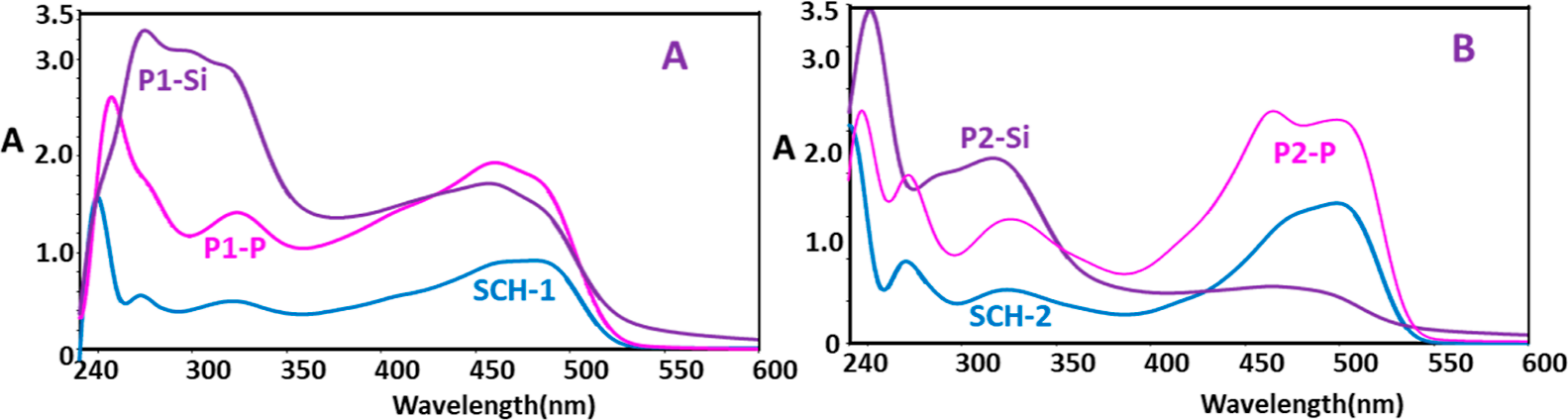
UV–vis absorption spectra of (A) SCH-1, P1–P, and P1–Si and (B) SCH-2, P2–P, and P2–Si.

The UV–vis spectra of the synthesized SCHs and oligo(azomethine)s are presented in Figure 5. UV–vis measurements conducted in DMSO provided valuable insights into the electronic transitions of the synthesized compounds. As observed in Figure 5, the spectra of SCH-1 and SCH-2 monomers displayed two absorption bands at 249 and 273; and 240 and 270 nm due to the delocalization of the aromatic electrons, indicative of the π → π* electronic transition. The third band at 321 nm and 326 nm is assigned to π → π* transition of the HC=N moiety of SCH-1 and SCH-2, respectively.^71^ 2-hydroxy naphthaldimines, presumed to act as intramolecular hydrogen bonding agents, e.g., O–H···N (phenol-imine tautomer) and O···H–N (keto-amine tautomer)^72^ displayed absorbance at 321 nm and 485 nm; 326 nm and 489 nm, for SCH-1 and SCH-2, respectively (Figure 5A and Figure 5B). These absorptions are associated with *n* → π* transitions of the C=N and C=O groups. Notably, the keto-amine tautomer consistently manifested when Schiff bases were derived from 2-hydroxynaphthaldehyde.^73^ The predominant tautomeric state of SCH-1 and SCH-2 in DMSO is the keto-amine tautomer. This is evidenced by the spectral bands in the region above 400 nm, which signify the presence of the keto-amine form, specifically associated with the *n* → π* transition of the carbonyl chromophore. In the spectra of SCH-2, the absorbance intensity exhibited a hyperchromic shift, attributable to the heightened likelihood of the keto form of SCH-2 being induced by the electron-donating −OCH_3_ group. Conversely, the FT-IR spectra of the Schiff base monomers revealed their predominant existence in the phenol-imine form in the solid state. This determination is supported by the absence of an observable peak at around 1700 cm^–1^, characteristic of the C=O stretching band.

A methodology can be outlined herein for distinguishing between tautomers of SCH-1 and SCH-2, relying on the identification of specific carbon types, namely, the carbonyl carbon (C=O) and phenolic carbon (=C–OH) atoms, through their respective ^13^C NMR chemical shifts. In the ^13^C NMR spectra of SCH-1 and SCH-2, the absence of peaks corresponding to carbonyl carbons (C=O) is noteworthy. Such peaks, characterized by their distinctiveness and typically situated at the low-field end of the spectrum within the chemical shift range of 180 to 220 ppm,^74^ were not detected. Furthermore, the absence of the singlet corresponding to the OH proton peak at 9.73 ppm and the presence of singlet peak at 8.78 ppm attributed to the azomethine CH=N proton suggests the incidence of the phenol-imine tautomer in SCH-1.^75^ The consistency of the phenol-imine tautomer in the solid state is corroborated by the FT-IR spectrum of the compound in question. Specifically, the presence of C=N stretching vibrations at 1611 and 1612 cm^–1^ indicates that the phenol-imine form is the predominant tautomer of SCH-1 and SCH-2 in the solid state.

In the spectra of P-oligo(azomethine)s and Si-oligo(azomethine)s, the π → π* electronic transitions of aromatic chromophores were observed to shift bathochromically, indicating an increase in conjugation during oligomerization. The *n* → π* transition of the P-oligo(azomethine)s also exhibited a hyperchromic shift in absorbance intensity within the range of 400–550 nm, attributed to the incorporation of P=O subsequent to the attachment of the phenyl dichlorophosphate unit to SCH-1 and SCH-2. In contrast, the *n* → π* transition associated with the CH=N group of Si-oligo(azomethine)s demonstrated a hypochromic shift in absorbance intensity. This phenomenon was attributed to the connection with the dichlorodiethylsilane unit on SCH-1 and SCH-2, as depicted in Figure 5A and Figure 5B. The synthesis of oligo(azomethine)s involved the elimination of HCl between the −OH group of the SCHs and the phenyl dichlorophosphate or dichlorodiethylsilane units, resulting in the formation of a terminal OH group.

A reduced bandgap in the oligomers facilitates electronic transitions between the highest occupied molecular orbital and lowest unoccupied molecular orbital energy levels, consequently enhancing higher electrical conductivity relative to the synthesized Schiff base of the oligomer. The synthesized oligomers exhibited lower bandgap values in comparison to their monomeric counterparts. Specifically, in DMSO, the bandgap (*E*_g_) values for compounds designated as SCH1, SCH2, P1–P, P1–Si, P2–P, and P2–Si were calculated to be 2.40, 2.30, 2.33, 2.30, 2.26, and 2.24 eV, respectively. The bathochromic shift resulted in a reduction of the bandgap energy (*E*_g_) for the oligo(azomethine)s. In light of these elucidations, it is apparent that P-oligo(azomethine)s and Si-oligo(azomethine)s exhibited favorable semiconductor properties, as evidenced by their lower *E*_g_ values in comparison to those of the monomers SCH-1 and SCH-2. The *E*_g_ values of P2–P and P2–Si were comparatively lower than those of P1–P and P1–Si. This disparity can be attributed to the presence of the electron-donating −OCH_3_ group, which augments the electron cloud distribution over the molecule.

**Figure 6:**
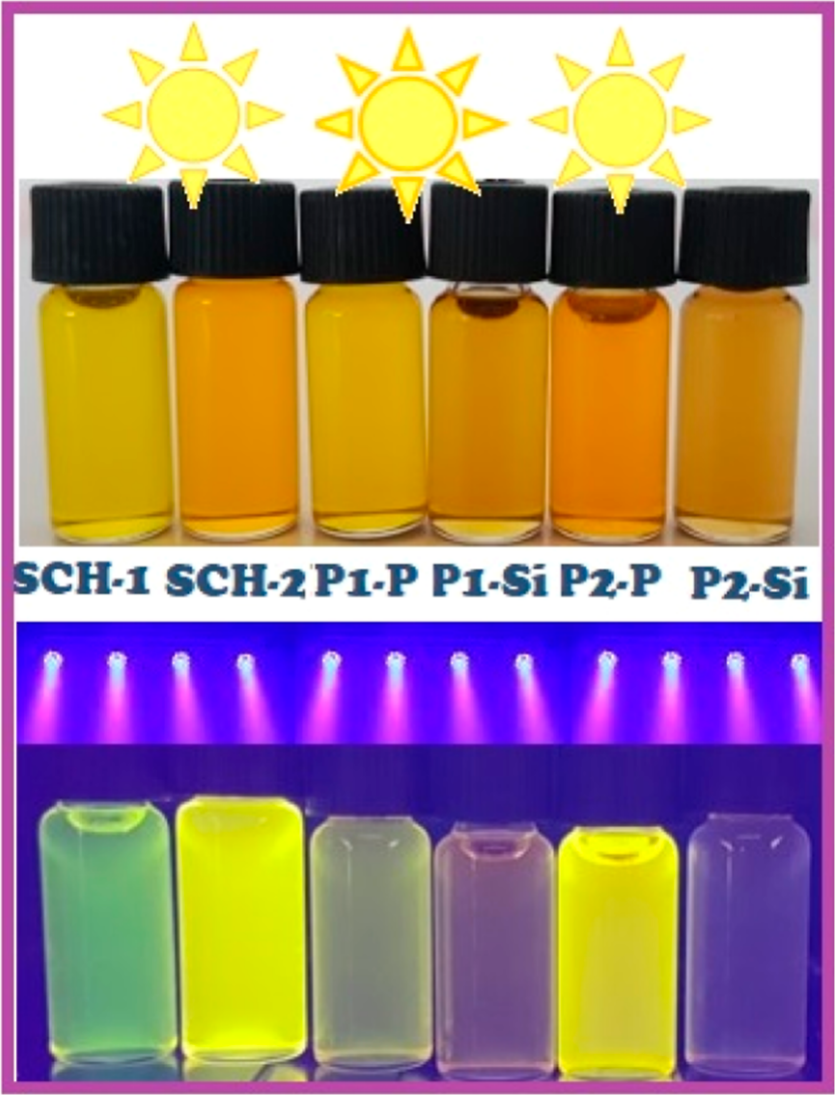
Photographs of SCH-1, SCH-2, and their respective P- and Si-oligo(azomethine)s under sunlight vs UV light (366 nm) in DMF.

Figure 6 depicts the observable chromatic attributes exhibited by the synthesized dihydroxy SCHs in conjunction with their oligo(azomethine)s when subjected to both sunlight and ultraviolet (UV) radiation. When exposed to UV light at 366 nm, the initially pale yellow and pale orange solutions of SCH-1 and SCH-2 underwent a transition in hue, resulting in a green and bright yellow appearance, respectively. The colors of P1–P and P2–P solutions turned light green and yellow, respectively. In contrast, Si–P1 and Si–P2 exhibited no discernible alteration in coloration when irradiated with UV light.

#### 3.4.2 PL Emission Properties

**Figure 7:**
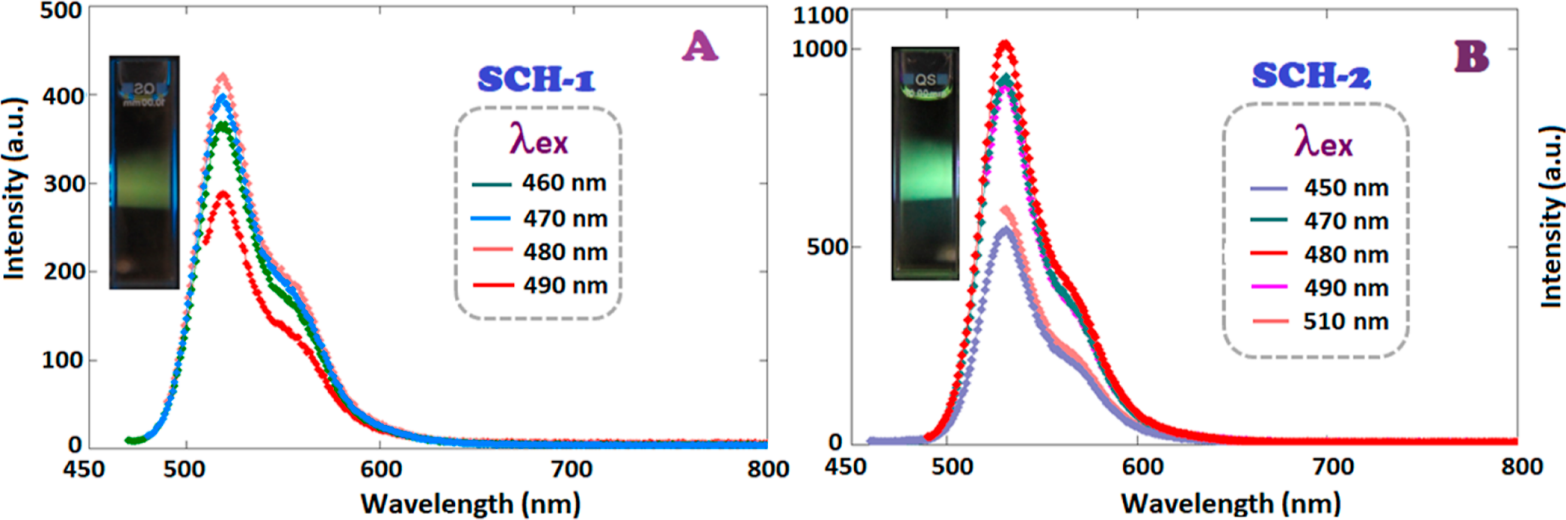
PL emission spectra of (A) SCH-1 and (B) SCH-2 in DMF (excitation and emission slit widths: 5 nm).

**Figure 8:**
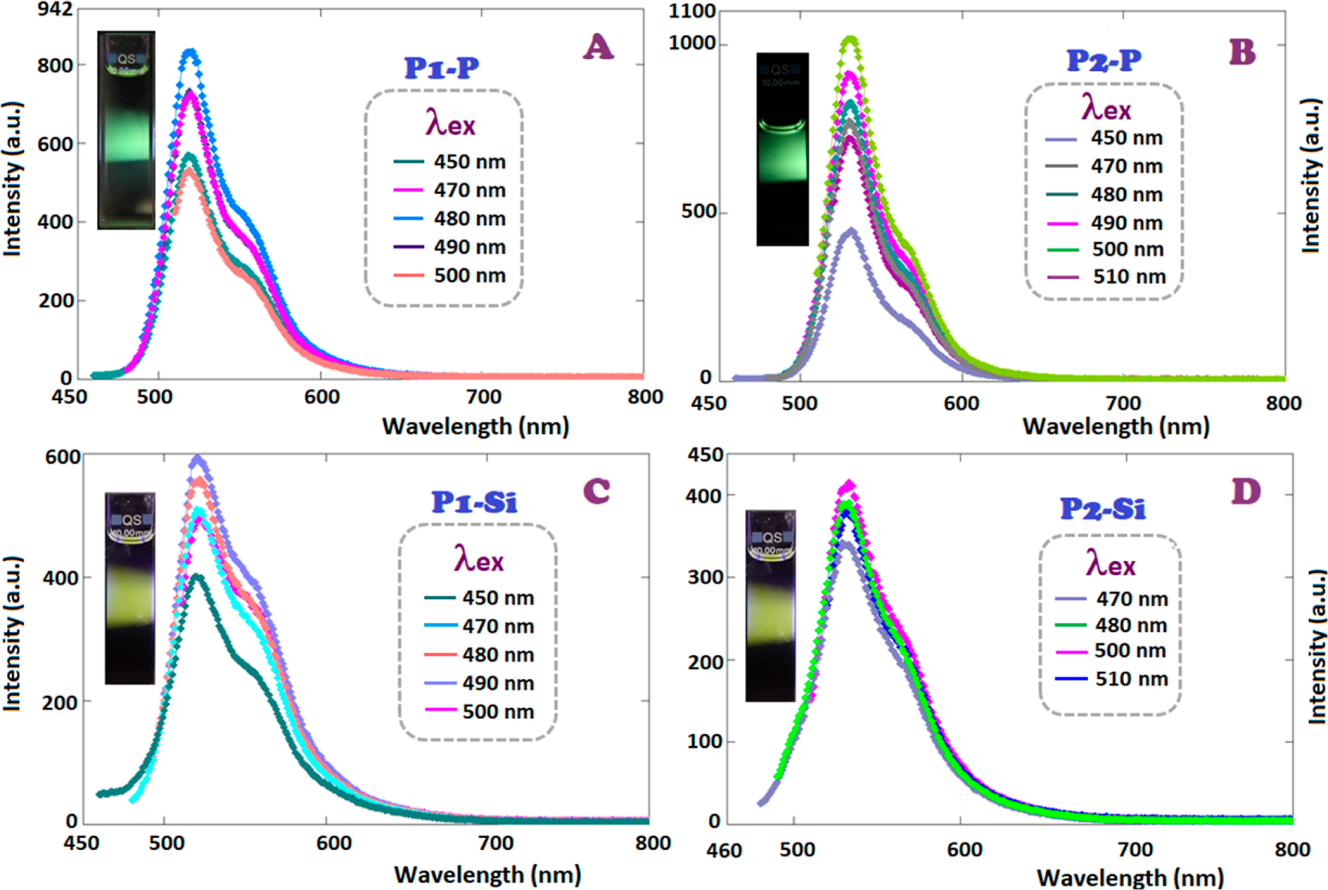
PL emission spectra of (A) P1–P, (B) P2–P, (C) P1–Si, and (D) P2–Si in DMF (excitation and emission slit widths: 5 nm).

The photoluminescent (PL) emission spectra of the synthesized SCHs and oligo(azomethine)s in DMF versus excitation wavelength and concentration are shown in Figure 7 and 8. When SCH-1 was excited with blue light at 470 nm, it yielded green light emission characterized by a peak intensity at 519 nm, with a recorded intensity of 421 a.u., as illustrated in Figure 7A. The formation of an intramolecular hydrogen bond between the nitrogen atom of the CH=N group and the −OH group has been proposed to significantly contribute to an increase in the fluorescence quantum yield. Intramolecular hydrogen bonding assumes a pivotal role in the proton transfer process. The heightened strength of hydrogen bonding in the excited-state molecules proves to be advantageous for the advancement of excited-state intramolecular proton transfer (ESIPT).^75,76^

SCH-2, characterized by the presence of a methoxy group, exhibited a different emission behavior compared to SCH-1, which contains a methyl group. Upon excitation at 470 nm, SCH-2 displayed a maximum emission wavelength at 530 nm. It emitted green light with a maximum emission intensity of 1022 a.u (Figure 7B).

The augmented acceptor strength resulting from the presence of the electron-donating −OCH_3_ group in SCH-2, concerning intramolecular hydrogen bonding, may elucidate the observed red-shifted photoluminescence spectrum. The intramolecular hydrogen bond experiences a notable increase in the excited state. Consequently, this enhancement proves advantageous for the intramolecular proton transfer process in the excited state.^77^

The ESIPT process arises from the enol–keto phototautomerization process. Under conditions of light excitation, for systems capable of undergoing the ESIPT process, electrons within the enol structure undergo a transition, absorbing light energy and moving from a lower-energy orbital to a higher-energy orbital. This transition is concomitant with the proton transfer process, resulting in the formation of a keto structure. However, electrons residing in the higher-energy orbital are inherently unstable and promptly transition back to the lower-energy orbital. Throughout this process, energy is emitted in the form of light. In cases where the system exhibits two emission peaks, these are expected to correspond to a stable enol structure and a stable keto structure, respectively.^78^

2-hydroxynaphthylidene-10-naphthylamine serves as an illustration of hydroxyderivatives within heteroaromatic molecules, commonly undergoing the ESIPT reaction, transitioning from the enol to keto tautomer. The elevated keto structure subsequently reverts to the ground state through the emission of fluorescence and/or experiences cis–trans isomerization. This process engenders the formation of the photochromic transient, ultimately culminating in the restoration of the initial enol structure, thereby completing the so-called proton transfer (PT) cycle.^79^

The emission spectrum’s shape of P1–P remained unaffected by variations in the excitation wavelength; however, an increment in excitation wavelength up to 480 nm led to an increase in emission intensity. The maximum emission (λ_em_) was observed at 519 nm, with a green light emission intensity of 840 a.u. upon exciting P1–P with 480 nm blue light (Figure 8A). The heightened intensity could be ascribed to the integration of P=O subsequent to bonding of the phenyl dichlorophosphate unit to SCH-1 and SCH-2. P2–P showed PL behavior similar to that of its Schiff base monomer. With an increase in conjugation, λ_em_ was measured at 532 nm when excited at 500 nm. A green light emission with a maximum intensity of 1016 a.u. was observed (Figure 8B).

**Figure 9:**
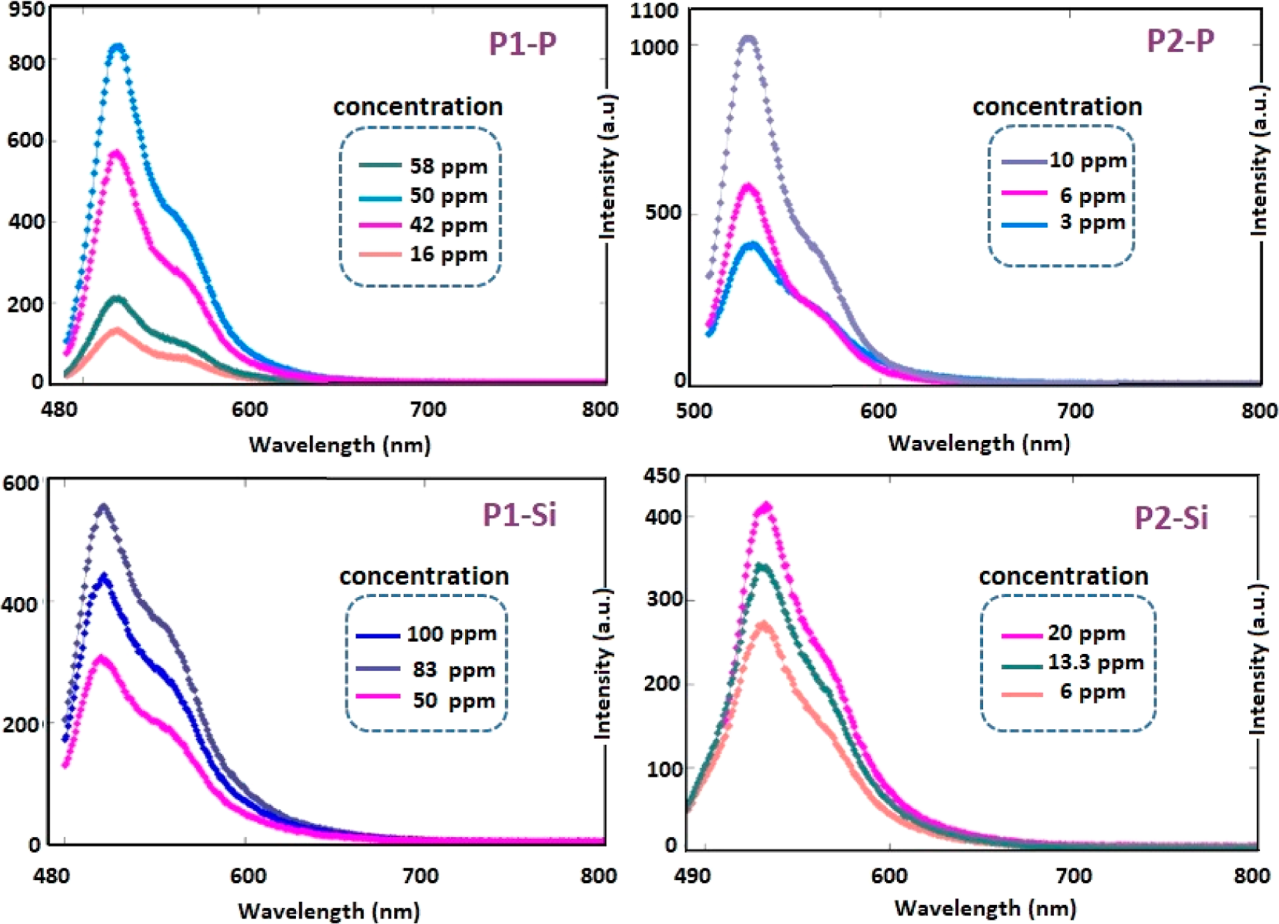
PL emission spectra of P1–P, P1–Si, P2–P, and P2–Si in DMF depending on concentration.

Similar emission behavior to P1–P and P2–P was observed for P1–Si and P2–Si, respectively. An increase in the excitation wavelength from 450 to 490 nm led to a green light emission for P1–Si at 521 nm, albeit with a diminished intensity of 593 a.u. compared to the maximum peak intensity exhibited by P1–P (Figure 8C). Correspondingly, upon excitation at 500 nm, P2–Si, unlike P2–P, emitted light at 532 nm with a reduced emission intensity of 412 a.u (Figure 8D). The diminished intensity may be attributed to the connection with the dichlorodiethylsilane unit on SCH-1 and SCH-2, leading to the disruption of conjugation along Si-oligo(azomethine)s. Fluorescence intensity of P1–P and P1–Si obtained an optimum value at 50 and 83 ppm, respectively, when excited at wavelength of 480 and 490 nm, respectively, leading to green light emissions. Subsequently, quenching emission was observed as the concentration increased, as depicted in Figure 9. The PL intensity of P2–P and P2–Si exhibited an optimal response at 10 and 20 ppm, respectively, when subjected to excitation wavelengths of 500 nm, manifesting as a green-colored emission process. Subsequently, a reduction in the emission intensity was observed as the concentration increased, as depicted in Figure 9. Extended conjugation ensured the stability of the excited state, enabling the oligomers to emit strongly. The light emitted nearly the same colors regardless of the applied excitation wavelength, owing to the absorption by numerous chromophores, including imine bonds, phosphate, and silane units.

The QYs for SCH-1, SCH-2, P1–P, P1–Si, P2–P, and P2–Si in DMF, corresponding to the spectra depending on maximum PL intensities in Figures 7 and 8, were found to be 2.1, 12.3, 16.2, 15.3, 16.1, and 2.8%, respectively.

**Figure 10:**
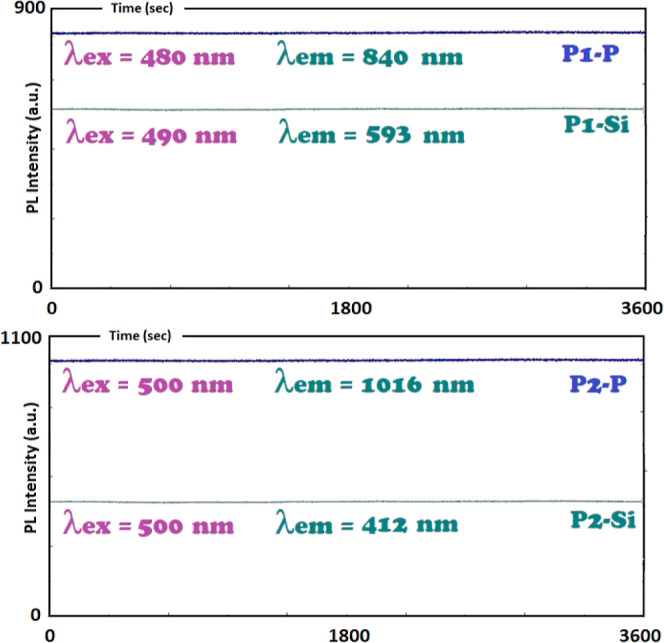
Time-dependent PL measurements of P-oligo(azomethine)s and Si-oligo(azomethine)s in DMF (excitation and emission slit widths: 5 nm).

Figure 10 presents time-dependent fluorescence measurements over a duration of 3600 s, contingent on the excitation wavelength employed. Within this specified time frame and under consistent experimental conditions, no discernible fluctuations in fluorescence intensity were observed. This observation provided a support for the constancy of photoluminescent (PL) emission in oligo(azomethine)s across varying excitation wavelengths. The excitation wavelengths of 480 nm for P1–P, 490 nm for P1–Si, and 500 nm for P2–P and P2–Si all resulted in persistent PL emission profiles over the 3600 s monitoring period. This outcome carries significance in the context of the potential suitability of the synthesized oligo(azomethine)s for application in light-emitting diodes (LEDs), as it underscores their strong performance in maintaining PL emission stability across a range of excitation wavelengths.

### 3.5 Thermal Analyses (TG-DTG-DSC)

**Figure 11:**
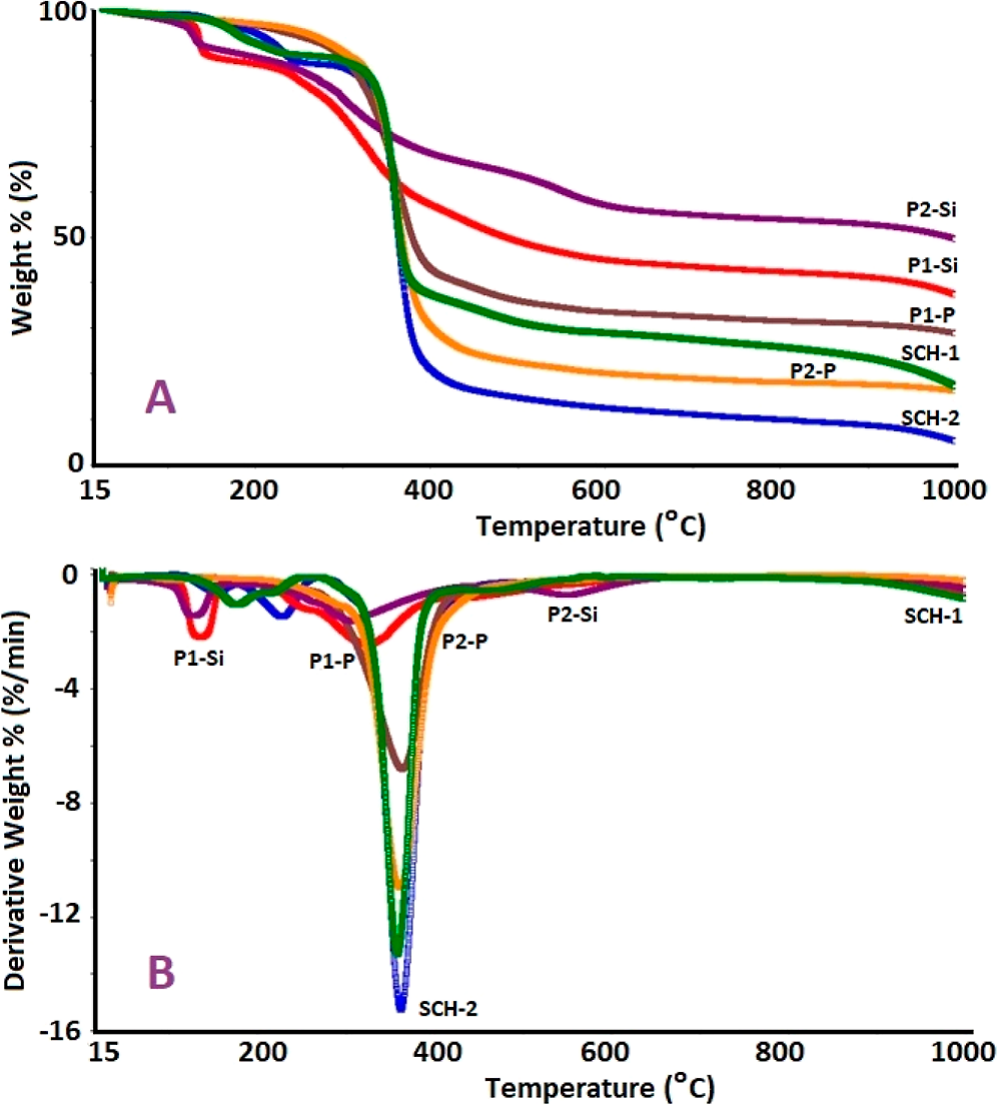
(A) TG and (B) DTG curves of the synthesized Schiff base monomers and oligo(azomethine)s.

The TG and DTG curves of the SCHs as well as their oligo(azomethine)s are presented in Figure 11. The values extracted from DSC, acquired to determine the glass transition temperatures (*T*_g_) and the corresponding heat capacity changes (Δ*C*_P_) during this transition, are summarized in Table 2. The TG curves provide information about the temperatures at which *T*_20_, *T*_50_, and *T*_on_, as well as the number of degradation stages, can be specified.

**Table 2:** Thermal Analysis Results of SCH-1, SCH-2, P-Oligo(azomethine)s, and Si-Oligo(azomethine)s

	SCH-1	SCH-2	P1–P	P1–Si	P2–P	P2–Si
a*T*_on_ (°C)	148	178	245	250	290	223
b*T*_max_ (°C)	178,359	229,363	363,468	325,445	360	308,549
*T*at 20% weight loss (°C)	346	340	338	329	342	330
*T*at 50% weight loss (°C)	366	364	384	940	369	
char % at 1000 °C	18.66	6.22	29.45	47.68	28.17	55.47
DSC [c*T*_g_ (°C)/dΔ*C*p (J/g)]	–/–	–/–	84/0.0469	142/0.026	88/0.146	126/0.048

a The onset temperature.

b Maximum weight loss temperature.

c Glass transition temperature.

d Change of specific heat during glass transition.

Figure 11A reveals an initial weight loss from 1.0 to 10.0%, primarily ascribed to the removal of volatile fragments, e.g., adsorbed water and solvent, up to a temperature of 100 °C.^80^ Based on the data summarized in Table 2, in comparison to SCHs, the synthesized oligomers, particularly the P-oligo(azomethine)s, exhibit higher *T*_on_ temperatures, indicating significantly enhanced thermal stability. The initial degradation temperatures (*T*_on_) for the SCHs and their corresponding oligo(azomethine)s were determined as follows: SCH-1 (148 °C), P1–P (245 °C), P1–Si (250 °C), SCH-2 (178 °C), P2–P (290 °C), and P2–Si (223 °C). Notably, the synthesized oligo(azomethine)s exhibited remarkable thermal stability up to 290 °C.

The DTG curves in Figure 11B indicated that the thermal degradation of SCH-1 and SCH-2 took place in two steps, with maximum decomposition temperatures (*T*_max_) of 178 and 359; and 229 and 363 °C, respectively. The DTG curves of oligo(azomethines)s exhibited a two-step degradation, with the exception of P2–P, which underwent a single-step degradation. P1–P exhibited weight losses of 58.56 and 10.99% in the temperature ranges 100–435 and 435–1000 °C, respectively. P1–Si lost 32.88 and 19.43% of its mass between 150 and 405; 405 and 1000 °C, respectively. Owing to the greater polarity of the Ar–O–P bond in comparison to the Ar–O–Si bond, the preferential cleavage of the diethylsilane group within P1–P over the cleavage of the phenyl phosphate group within P1–S is observed in the initial decomposition stage. Subsequently, the second decomposition step entails cleavage of the imine (C=N) bond present in P1–P and P1–Si. The DTG curve for P2–P revealed a single-step degradation process with a maximum decomposition temperature of 360 °C. Notably, the cleavage of the –OCH_3_ group from the aromatic ring occurs more readily in comparison to the –CH_3_ group, thereby resulting in the concurrent occurrence of both Ar–O–P and –OCH_3_ bond cleavages within a single-step degradation process in P2–P. In contrast, for P2–Si, a two-step decomposition profile was observed, with the first stage occurring between 130 and 453 °C, eventuating with a mass loss of 28.31%, followed by the second stage within the temperature range of 453–1000 °C, resulting in a mass loss of 16.22%.

The DSC measurements revealed *T*_g_ values for P1–P, P2–P, P1–Si, and P2–Si as 84, 88, 142, and 126 °C, respectively. Due to the greater polarity exhibited by the P–O bond in contrast to the Si–C bond, the cleavage of the P–O bond within the P1–P and P2–P chains is anticipated to occur more readily than that of the Si–C bond. Consequently, this disparity in bond cleavage contributes to elevated glass transition temperatures (*T*_g_) observed in Si-oligo(azomethine)s in comparison to P-poly(azomethine)s.

The char % yield increased from 28.66% of SCH-1 to 29.45% of P1–P and 47.68% of P1–Si. Similarly, the same trend was observed for SCH-2 from 6.22 to 28.17% for P2–P and to 55.47% for P2–Si at 1000 °C, indicating that the incorporation of P and Si atoms used as flame retardants significantly enhanced the char yields significantly. A higher char yield substantially augments flame retardance. An increase in the formation of char brings about several beneficial effects, including the inhibition of the production of flammable carbon-containing gases and a reduction in the thermal conductivity of the burning material’s surface.^81^ Van Krevelen established a linear correlation between the limiting oxygen index (LOI), which is used to assess the material flammability, and the quantity of char residue in oligomers.^82^ The calculated LOI values for P1–P, P2–P, P1–Si, and P2–Si were 29.28, 28.77, 36.57, and 39.69, respectively. All of these values exceeded the threshold value of 26, thereby endowing them with self-extinguishing properties.^83,84^ This disparity highlights the superior flame-retardant characteristics of Si-oligo(azomethine)s in comparison to P-oligo(azomethine)s.

Additionally, the heat resistance index (*T*_HRI_) was also computed following the method outlined by Satdive et al.^85^ and Arora et al.,^86^ utilizing the temperatures corresponding to 5 and 30% weight loss (*T*_5_ and *T*_30_). The calculated values for T_HRI_ of P1–P, P2–P, P1–Si, and P2–Si were 156.51, 158.86, 158.96, and 192.86, respectively. The incorporation of silicon element is evident in the enhancement of LOI and Thermogravimetric Analysis, indicating a notable improvement in both the thermal stability and flame retardancy of Si-oligo(azomethine)s. It can be argued that Si-oligo(azomethine)s, characterized by both high thermal stability and substantial char yield, hold potential utility in the fabrication of heat-resistant materials.

### 3.6 Morphological Characteristics of Oligo(azomethine)s and XRD Analysis

**Figure 12:**
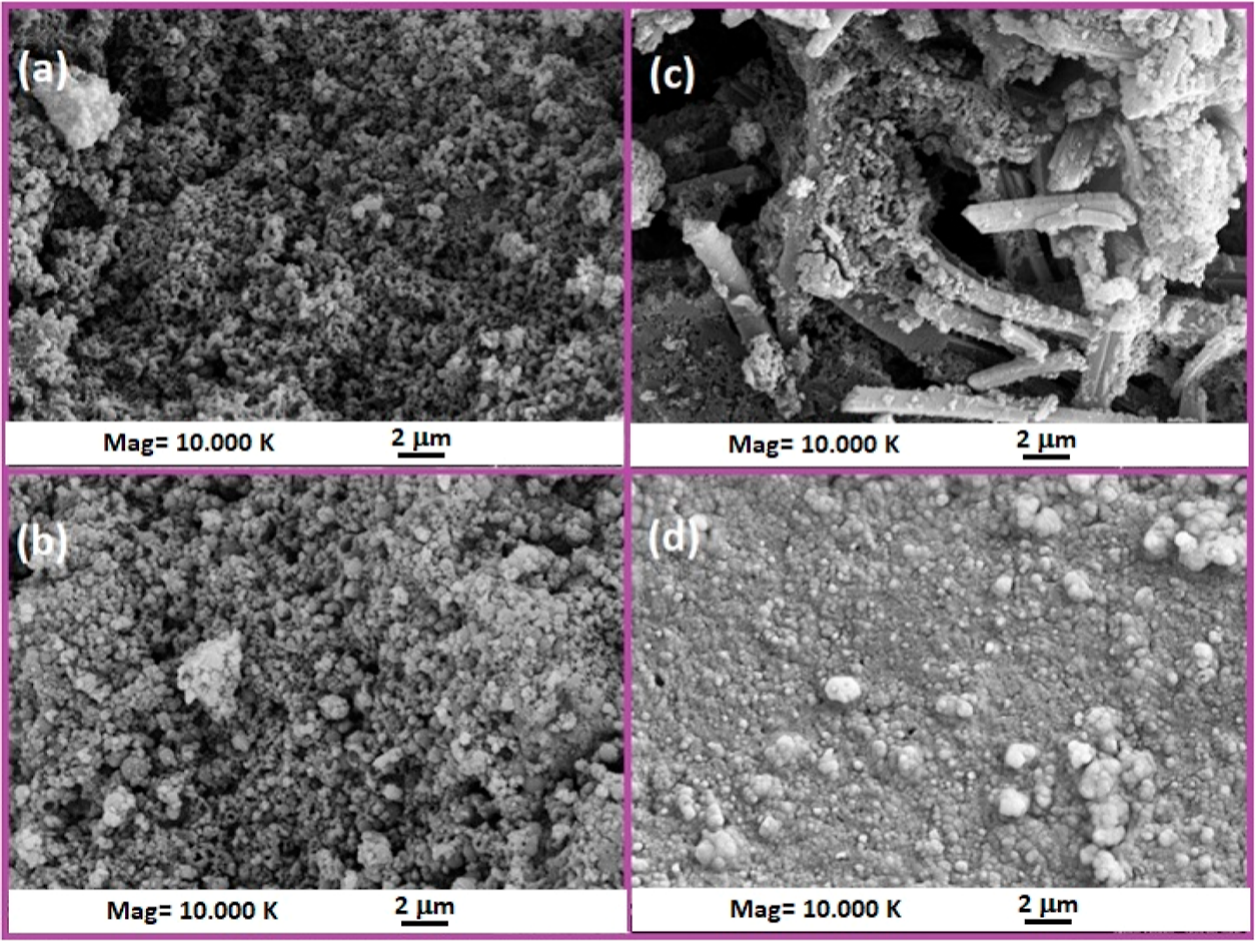
SEM photographs of (a) P1–P, (b) P1–Si, (c) P2–P ve, and (d) P2–Si.

The morphological characteristics of the synthesized oligo(azomethine)s were analyzed by using SEM images. SEM images of P1–P, P1–Si, P2–P, and P2–Si are presented in Figure 12. Surface properties were observed to vary depending on the inclusion of diethylsilane (Si) and phenyl phosphate (P) units in the molecular structure of oligo(azomethine). As shown in Figure 12a,b, the surface morphologies of P1–P and P1–Si revealed the clustering of particles at different nanometer scales. In Figure 12c, rod-like structures with dimensions of 2 μm were observed for P2–P. By way of contrast, the SEM image clearly demonstrated that there were no pores on the surface of P2–Si, as seen in Figure 12d. P2–Si presented a more compact surface; such a continuous and thermally stable structure can act as a protective barrier, as an efficient shield and insulation thus preventing the sample from fire and heat.^87^

The XRD patterns for SCH-1, SCH-2, and their oligo(azomethine)s are depicted in Figures S4 and S5, and the corresponding crystallite sizes (*D*) for the monomer and oligomer are presented in Table S1. The calculated (*D*) values provide insights into the crystal structure types and interlayer spacing (d). Prior to analysis, the SCH-1, SCH-2, and oligo(azomethine) samples underwent meticulous grinding and homogenization. Utilizing the diffraction data in Table S1, the mean crystallite sizes (*D*) of the monomer and polymer were determined using the Debye-Scherer equation: *D* = (0.9λ)/(β.cos θ), where λ represents the X-ray wavelength (1.5406 Å), θ is the Bragg diffraction angle, and β is the full width at half-maximum of the diffraction peak.^88^

Considering the significance of ascertaining crystal sizes and interlayer spacings (d) derived from the diffraction angle of the corresponding peaks in the compounds, the average crystallite size of the synthesized Schiff bases and their oligo(azomethine)s was determined. Analysis of the XRD patterns within the 2θ range from 5 to 80° revealed highly intense diffraction peaks at 2θ = 11.80, 12.11, and 25.50°, indicating that a well-crystallized structure for SCH-1 was formed. Additionally, two less intense peaks were observed at 2θ = 27.48 and 32.80° for SCH-1, as illustrated in Figure S4, indicating multiple crystals in one particle. Subsequently, the peaks corresponding to SCH-1 exhibited decreased intensity and broadening in P1–Si, suggesting the amorphous nature of the sample.^89^ In the case of P1–P, the peaks exhibit attenuation and broadening. The calculated average crystallite sizes for SCH-1, P1–Si, and P1–P were 93.95 nm, 16.30 nm, and 32.80 nm, respectively. Notably, a distinct sharper peak appeared at 2θ = 18.30° for P1–P, indicative of a relatively larger crystal with a size of 32.80 nm. Conversely, the observed broader peak and smaller crystallite size of 16.30 nm in P1–Si suggests that P1–Si may exhibit in an amorphous nature.^90^

Similar to SCH-1, sharp peaks with high intensity were evident at 2θ = 7.67, 12.90, and 26.51° for SCH-2, accompanied by two less intense peaks at 2θ = 9.77 and 16.65°, indicating multicrystallinity within a single particle, as depicted in Figure S5. In contrast, P2–Si exhibited broadened peaks, indicative of the loss of crystallites and the formation of amorphous material. Furthermore, the multiple peaks of P2–P, with a lower intensity compared to SCH-2, suggest a multicrystalline nature of the material. The calculated average crystallite sizes for SCH-2 and P2–P are 99.02 and 53.46 nm, respectively, with the larger crystallite size attributed to the influence of high crystallinity. On the other hand, the existence of a broader peak and a smaller crystallite size of 16.22 nm in the P2–Si sample suggests the potential manifestation of an amorphous nature within this sample. As a result, SCH-1 has a larger crystallite size compared to SCH-2, potentially due to the presence of the –OCH_3_ group which impedes the formation of larger crystallites. Similarly, P1–P demonstrates a larger crystallite size than P2–P, possibly due to the presence of –OCH_3_ group that hinders particles from forming larger crystallite size. The smaller crystallite size observed in Si-oligo(azomethine) compared to that in P-(oligo)azomethines could be the presence of more flexible diethylsilane groups along the oligomer chain, resulting in an amorphous nature within Si-oligo(azomethine) structures.

## 4 Conclusions

The primary objective of this investigation was to synthesize conjugated oligomers, specifically silane- and phosphate-based ones, denoted as P1–Si, P2–Si, P1–P, and P2–P, all derived from the bis-azomethine Schiff bases. Employing UV–vis analysis, changes in the solvent polarity did not significantly affect the π → π* electronic transition of the synthesized oligo(azomethine)s. P-oligo(azomethine)s emerged as a promising candidate for constructing materials designed for stable semiconductor-based green light irradiation sources. Notably, no significant changes in fluorescence were observed over a duration of 3600 s under identical conditions. PL quantum yield values obtained in DMF ranged from 2.8 to 16.2%, depending on the molecular structure of the oligomers, suggesting their applicability in optical and electronic devices. In both absorption and emission spectra, the oligo(azomethine)s containing a methoxy group exhibited a red shift when compared to the oligo(azomethine)s with methyl groups. This observed bathochromic shift could be anticipated owing to the greater electron-donating nature of the –OCH_3_ functional groups relative to the –CH_3_ moieties. Furthermore, Si- oligo(azomethine)s, characterized by high char values of 47.68 and 55.47%, demonstrated greater thermal stability compared to P-oligo(azomethine)s. This distinction suggested the potential utility of Si-oligo(azomethine)s in the production of heat-resistant materials. SEM images revealed the absence of pores on the surface of P2–Si, which exhibited elevated LOI and Thermal Heat Release Index (*T*_HRI_) values. This observation indicates a more compact surface, conducive to a thermally stable structure, thereby mitigating the susceptibility of the sample to fire and heat. In forthcoming investigations, the synthesis of oligo(azomethine)s will be pursued with the objective of enhancing flame retardancy and heat resistance properties. Moreover, the luminescent characteristics of oligo(azomethine)s can be modulated through manipulation of the conjugation length and variation of substituents.

## Supporting Information Available

^1^H and ^13^C NMR of SCH-2 and its P- and Si-oligo(azomethine)s; and XRD patterns of SCH-1, P1–Si, P1–P, SCH-2, P2–Si, and P2–P as well as the tabulated XRD parameters (PDF)
