# Supplementary material for: Comparative Study of Bis-Schiff Case Containing Conjugated Oligomers Based on Phosphate and Silane Moieties: Investigation of Photophysical and Thermal Properties
Source: ACS Omega. 2024 May 28;9(23):24789–806. doi: 10.1021/acsomega.4c01403 (PMC11170720; doi:10.1021/acsomega.4c01403)
Supplement: Supplementary file 1 — ao4c01403_si_001.pdf [file ao4c01403_si_001.pdf]

Supporting Information

Comparative Study of Bis-Schiff Case Containing Conjugated Oligomers Based on Phosphate and Silane Moieties: Investigation of Photophysical and Thermal Properties

Feyza Kolcu[a,b], Süleyman Çulhaoğlu[a,c], İsmet Kaya[a*]

[a]Çanakkale Onsekiz Mart University, Department of Chemistry, Polymer Synthesis and Analysis Lab., 17020, Çanakkale, Turkey
[b]Çanakkale Onsekiz Mart University, Lapseki Vocational School, Department of Chemistry and Chemical Processing Technologies, 17800, Çanakkale, Turkey
[c]Barem Packaging Industry and Trade A.S., 35910, İzmir, Turkey

List of Contents:

Figure S1. ^{1}H and ^{13}C NMR of SCH-2
Figure S2. ^{1}H and ^{13}C NMR of P2-P
Figure S3. ^{1}H and ^{13}C NMR of P2-Si
Figure S4. XRD patterns of SCH-1, P1-Si and P1-P
Figure S5. XRD patterns of SCH-2, P2-Si and P2-P
Table S1. XRD parameters of SCH-1, SCH-2, Si-oligo(azomethine)s and P-oligo(azomethine)s

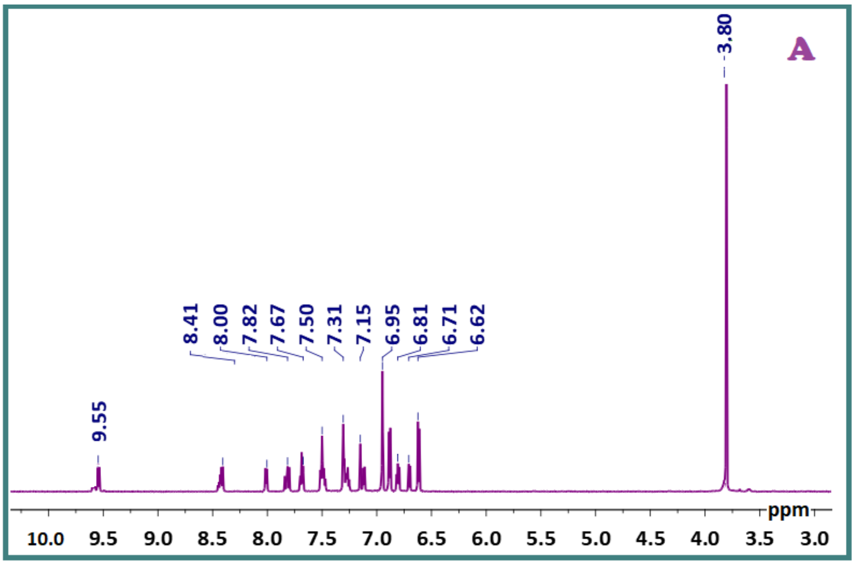

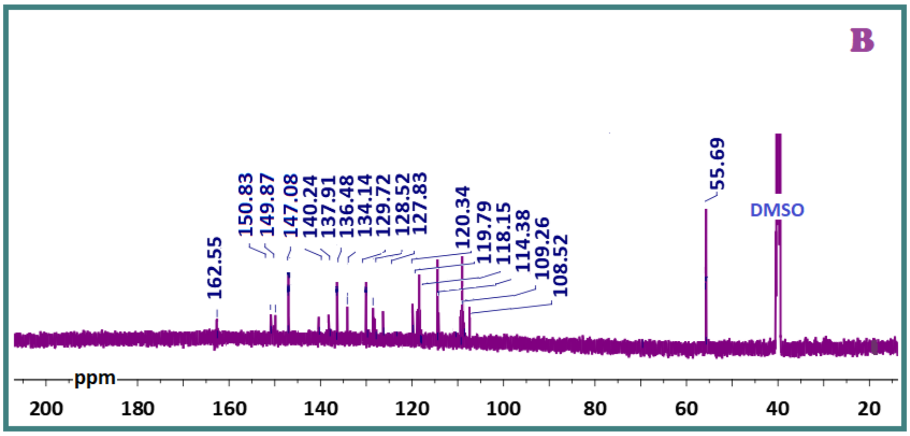

Figure S1. (A) ^{1}H and (B) ^{13}C NMR of SCH-2

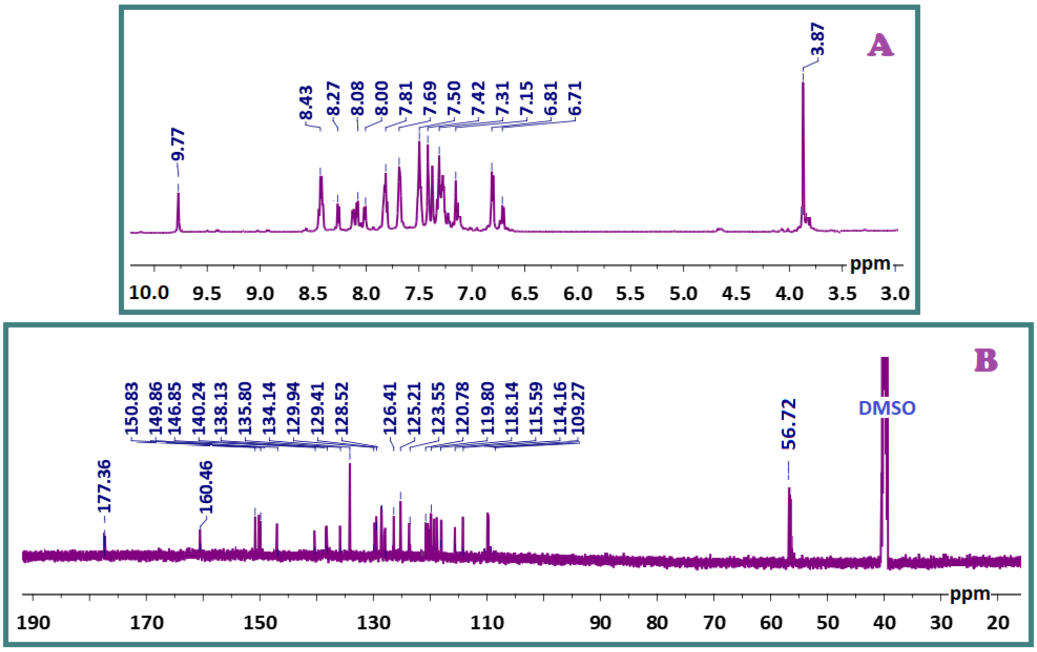

Figure S2. (A) ^{1}H and (B) ^{13}C NMR of P2-P

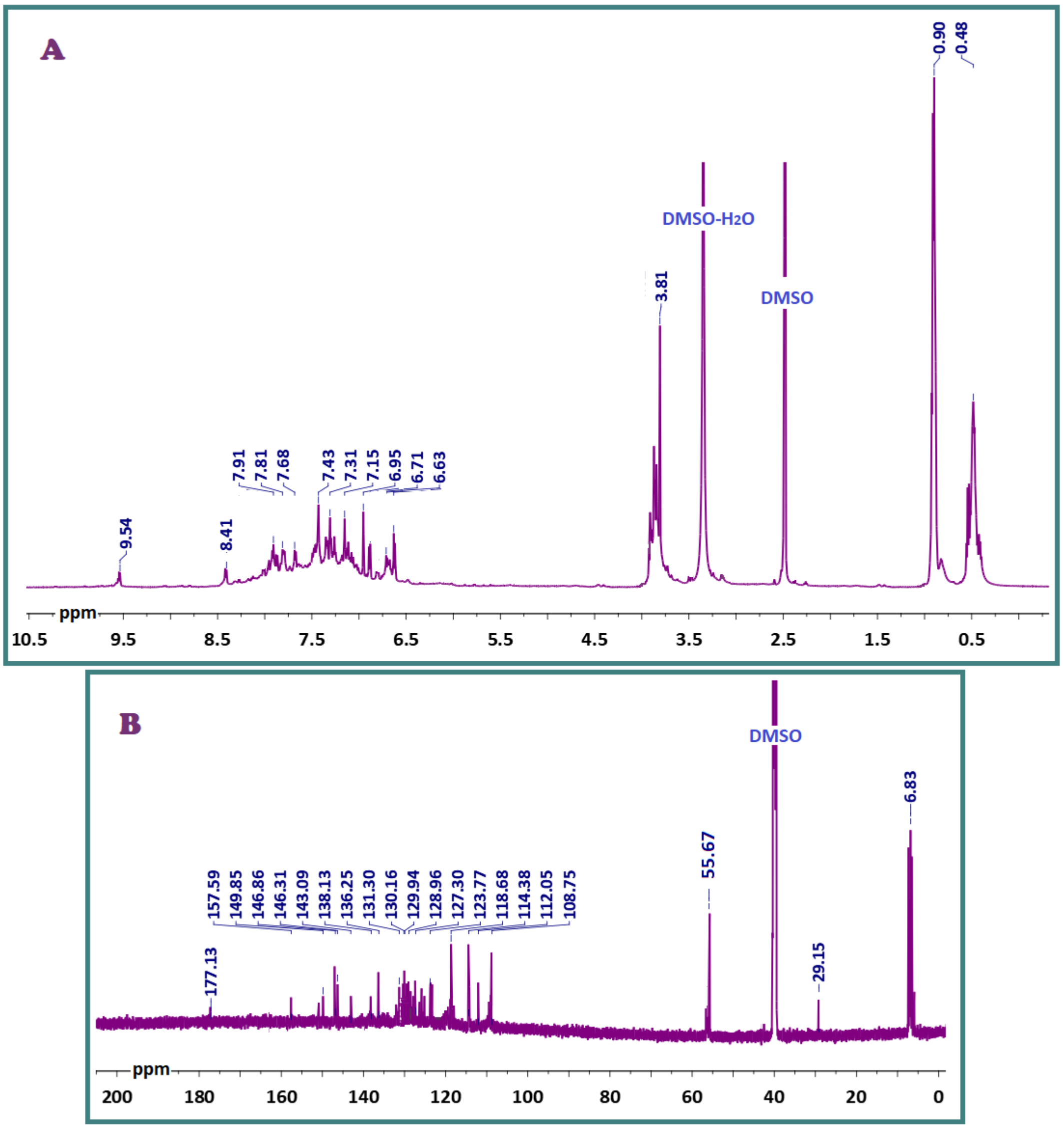

Figure S3. (A) ^{1}H and (B) ^{13}C NMR of P2-Si

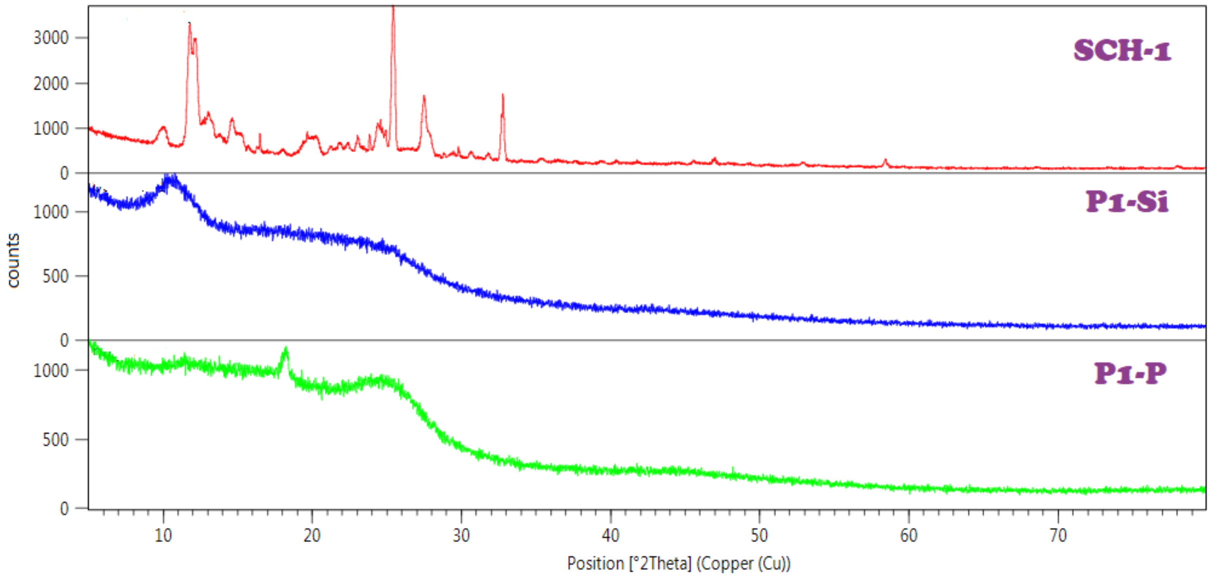

Figure S4. XRD patterns of SCH-1, P1-Si and P1-P

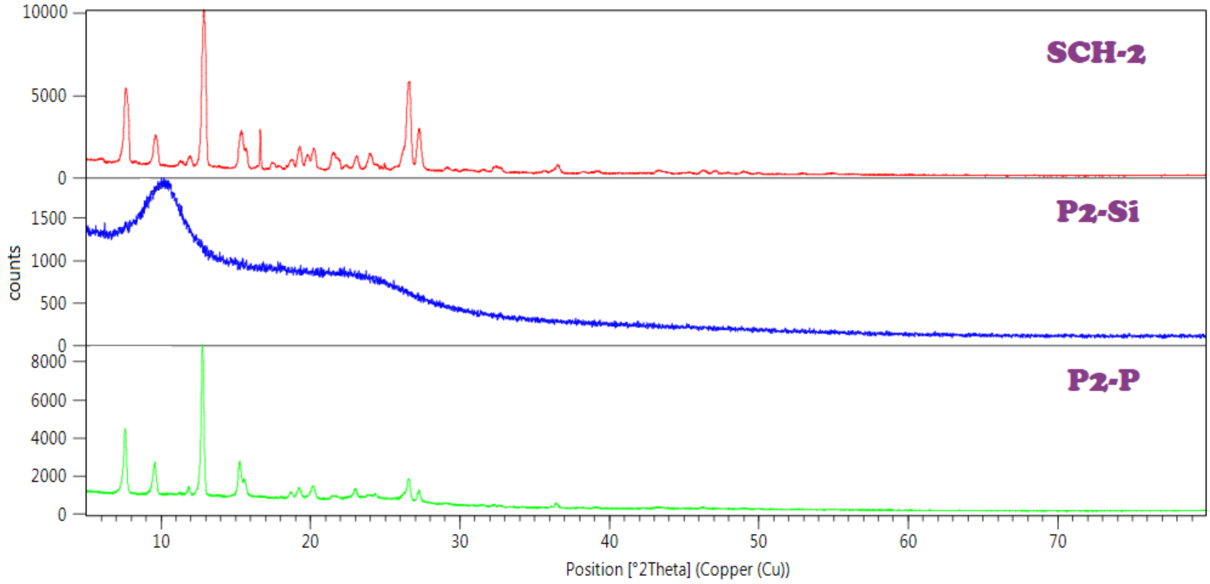

Figure S5. XRD patterns of SCH-2, P2-Si and P2-P

Table S1. XRD parameters of SCH-1, SCH-2, Si-oligo(azomethine)s and P-oligo(azomethine)s

Major peaks	Peak pos. [°2θ]	β [°2θ]	Cos θ	Crystallite size [*nm*]	d-spacing [*Å*]
**SCH-1**	11.80	0.1023	0.995	81.76	7.49950
	12.11	0.0768	0.994	109.4	7.30895
	25.50	0.1919	0.975	44.37	3.49380
	27.48	0.0895	0.971	95.52	3.24522
	32.80	0.0624	0.959	138.72	2.72829
**P1-Si**	10.91	0.5117	0.995	16.30	8.11205
**P1-P**	18.30	0.2558	0.987	32.88	4.84917
**SCH-2**	7.67	0.0768	0.997	108.42	11.52442
	9.77	0.0640	0.996	130.23	9.04957
	12.90	0.0640	0.993	130.62	6.86240
	16.65	0.0640	0.989	131.15	5.32323
	26.51	0.2184	0.973	39.06	3.36002
	26.66	0.1560	0.973	54.69	3.34080
**P2-Si**	10.17	0.5117	0.996	16.22	8.11205
**P2-P**	7.58	0.1151	0.998	7.227	11.65730
	9.58	0.1407	0.996	59.23	9.22720
	12.79	0.1279	0.994	65.29	6.92042
	15.27	0.1535	0.991	54.57	5.80382
	26.63	0.1023	0.973	83.40	3.34743
